# Global burden of disease changes related to high red meat diets and breast cancer from 1990 to 2021 and its prediction up to 2030

**DOI:** 10.3389/fnut.2025.1586299

**Published:** 2025-06-04

**Authors:** Yujun Tong, Hong Ning, Zhen Zhang, Xiaohong Zhang, HongMei Tu, Min Yang, Xiaoan Li, Tiantian Liang

**Affiliations:** ^1^Department of Breast Center, Mianyang Central Hospital, School of Medicine, University of Electronic Science and Technology of China, Mianyang, China; ^2^Department of Pharmacy, Mianyang Central Hospital, School of Medicine, University of Electronic Science and Technology of China, Mianyang, China; ^3^NHC Key Laboratory of Nuclear Technology Medical Transformation, Mianyang Central Hospital, School of Medicine, University of Electronic Science and Technology of China, Mianyang, China

**Keywords:** breast cancer, high red meat diet, global burden of disease, future trends, epidemiology

## Abstract

**Background:**

Breast cancer associated with high red meat consumption has become a significant global health issue. This study aims to analyze the global and regional disease burden related to breast cancer attributable to high red meat diets from 1990 to 2021, and to predict future trends in disease burden through 2030, providing scientific evidence for the development of targeted public health strategies.

**Methods:**

Data were extracted from the Global Burden of Disease (GBD) database, focusing on breast cancer-related attribution indicators, including the age-standardized rates (ASRs) of mortality, years of life lost (YLLs), years lived with disability (YLDs). The study analyzed the changes in breast cancer disease burden associated with high red meat consumption from 1990 to 2021 at the global level, across 21 regions, and in 204 countries. Future trends were projected using the Bayesian Age-Period-Cohort (BAPC) model.

**Results:**

In 2021, breast cancer deaths attributable to excessive red meat diets totaled 81,506, with YLLs amounting to 2,135,620 person-years and YLDs accounting for 214,442 person-years. These values represent increases of 80.83, 72.69, and 65.37%, respectively, compared to 1990. Despite global decreases in the ASRs of mortality and YLLs (which decreased to 1.15/100,000 and 30.12/100,000, with EAPCs of −0.77 and −0.73, respectively), the ASR of YLDs remained relatively stable (EAPC of −0.12). Stratification by Socio-Demographic Index (SDI) revealed a significant decline in disease burden in high-SDI regions, while the ASR in low-SDI regions trended upward. Projections suggested that by 2030, the global ASR of breast cancer burden may stabilize, while the burden in low-SDI regions is expected to continue rising.

**Conclusion:**

From 1990 to 2021, the global age-standardized rate of the breast cancer disease burden decreased, but disparities between regions with different SDI levels remain a major challenge. In the future, it is essential to prioritize addressing the burden in low-SDI regions and developing targeted interventions to optimize health resources, thereby mitigating the public health threat of breast cancer.

## 1 Introduction

In 2024, the International Agency for Research on Cancer (IARC) released the latest GLOBOCAN 2022 database, which encompasses data on the incidence and mortality of 36 types of cancer across 185 countries or regions worldwide. Breast cancer remains the most prevalent and deadliest malignancy among women globally, with the highest standardized incidence rate (SIR) and standardized mortality rate (SMR) among all cancers. It accounts for 11.6% of all cancers worldwide, approximately 1/4 of all newly diagnosed cancer cases in women, and 1/6 of cancer-related deaths, posing a significant threat to women’s health and lives (1).

Increasing evidence suggests that dietary factors play a crucial role in the incidence and mortality of breast cancer (2). Notably, in 2015, IARC classified fresh red meat as a possible carcinogen to humans based on a comprehensive expert evaluation (3). Red meat refers to the muscle tissue of mammals, including beef, pork, lamb, and others. It is rich in protein, fats, vitamins, and trace elements, serving as an important source for maintaining human health and development. However, it also contains high levels of saturated fat, nitrosamines, and other potential carcinogens (4). Excessive consumption of red meat has long been confirmed to increase the risk of cardiovascular diseases (5). In an umbrella review, it was found that a 100 g/day increase in red meat intake and a 50 g/day increase in processed meat intake were associated with 11–51% and 8–72% higher risks of multiple cancer outcomes (6). Meanwhile, several studies have also explored the relationship between red meat consumption and the risk of breast cancer (7). As early as 2006, researchers suggested that higher red meat intake might be a risk factor for ER+/PR + breast cancer in premenopausal women (8). In 2015, researchers reported a significant association between red meat consumption and an increased risk of breast cancer in a prospective cohort study involving 61,476 French participants (9). In 2020, Jamie J. Lo found that increased red meat consumption was associated with a higher risk of invasive breast cancer (10). Subsequently, Maryam S. Farvid conducted a systematic review on red meat consumption and various types of cancer, and similarly found that high red meat intake was significantly associated with an increased risk of breast cancer (RR = 1.09; 95% CI = 1.03–1.15) (7). However, the findings of related studies remain somewhat controversial, with some research suggesting that there is no significant association between red meat consumption and breast cancer (11, 12).

To address potential future health challenges, we systematically analyzed trends in breast cancer incidence and its association with dietary factors using data from the Global Burden of Disease (GBD) database. GBD is a comprehensive epidemiological study that provides up-to-date data on 371 diseases and injuries across 204 countries and territories. It also offers a thorough assessment of the impact of exposure to risk factors, categorized into four levels, on specific health outcomes (13). To compensate for the lack of health data in certain countries and regions and to improve the accuracy of estimates, the GBD study employs the Bayesian Age-Period-Cohort (BAPC) model. This approach decomposes trends from the perspectives of age, period, and cohort, while using a Bayesian framework to address data uncertainty and missing values. This analytical approach can integrate data from different regions and populations, identify the main drivers of disease and mortality, and forecast future trends (13, 14).

With ongoing changes in dietary patterns and population structures across countries, predicting the breast cancer burden and its relationship with high red meat consumption by 2030 has significant public health implications. Based on the GBD database, we calculated and utilized the age-standardized rates (ASRs) of mortality, years of life lost (YLLs), years lived with disability (YLDs) to analyze the breast cancer burden associated with high red meat diets. This analysis was conducted globally, by country, region, and Socio-Demographic Index (SDI) level, as well as by gender, and also included projections for trends up to 2030. This analysis will provide valuable insights for policymakers and public health researchers, aiding in the development of more effective prevention strategies and dietary recommendations to reduce the incidence and mortality of breast cancer.

## 2 Methods

### 2.1 Data source

The GBD study, conducted by the Institute for Health Metrics and Evaluation (IHME), provides comprehensive and up-to-date epidemiological analyses of disease and injury burdens across 204 countries and regions, covering 371 distinct conditions^1^ (15). The analysis draws on data from 100,983 sources, including censuses, household surveys, demographic records, disease registries, healthcare utilization data, environmental air quality measurements, and satellite imagery. These diverse data sources are categorized by country, region, age group, and gender, with comparability ensured through standardized coding and quality weighting.

All data undergo a rigorous process of standardization, cleaning, modeling, and adjustment. Consistency is validated using Bayesian meta-regression tools (DisMod-MR 2.1) and the Cause of Death Ensemble Model. To minimize regional bias, the analysis includes stratified assessments by the SDI, ensuring internal consistency of estimates across different regions, age groups, genders, and years. This methodology aims to mitigate the impact of heterogeneity on research outcomes through careful standardization and calibration (16, 17).

In the GBD 2021 study, the 204 countries and regions were classified into five levels of development based on their SDI scores. The SDI ranges from 0 to 1, with higher scores indicating higher socio-economic status. The SDI categories are as follows: low SDI (0 to <0.46), low-middle SDI (0.46 to <0.61), medium SDI (0.61 to <0.69), high-middle SDI (0.69 to <0.81), and high SDI (0.81 to 1.00) (18). Additionally, the world is divided into 21 GBD regions based on socio-economic and geographical conditions (19, 20).

### 2.2 Disease definition and data extraction

Breast cancer is a malignant tumor characterized by the abnormal proliferation and invasive growth of epithelial cells within breast tissue. Its diagnosis is established based on the TNM classification system, as defined by the Union for International Cancer Control (UICC) or the World Health Organization (WHO) classification of breast tumors (21). The etiology of breast cancer is multifactorial, involving a complex interplay of genetic predisposition (such as mutations in BRCA1/2 and other high-risk genes), prolonged exposure to estrogen and progesterone, lifestyle-related factors, environmental endocrine disruptors, and reproductive and breastfeeding patterns (22, 23).

According to the GBD 2021 study, breast cancer has become the most commonly diagnosed malignancy among women and is one of the leading causes of cancer-related disability and mortality, posing significant public health and socioeconomic challenges worldwide (24).

### 2.3 Dietary risk definition and data extraction

Within the four-level risk factor classification system of the GBD framework, high red meat diets are categorized as a Level 3 risk factor (19). A high red meat diet is defined as the consumption of more than 23 grams per day (range: 18–27 g/day) of unprocessed red meat—including beef, pork, lamb, and goat—while excluding poultry, fish, eggs, and all processed meats (3, 25).

We extracted population-attributable fractions (PAFs) for breast cancer associated with high red meat diets from the GBD 2021 database, which includes exposure distributions and corresponding risk estimates. Specifically, we obtained data on breast cancer-related deaths, YLLs, and YLDs associated with high red meat intake as key indicators for analysis. These metrics reflect both premature mortality and quality-of-life loss, providing a comprehensive evaluation of disease burden. Additionally, the SDI was extracted to assess the influence of socioeconomic factors on disease burden. All estimates were reported with 95% uncertainty intervals (UIs), including the mean, lower, and upper bounds.

### 2.4 Burden estimation

Given the heterogeneity in population age structures across regions and over time, age standardization is essential for comparability. ASRs were calculated using the direct standardization method based on the global age structure. All estimates were presented with 95% UIs, defined as the 2.5th and 97.5th percentiles of 1,000 ordered draws from the posterior distribution.


$$\text{ASR}=\frac{\sum_{i=1}^{A}a_{i}w_{i}}{\sum_{i=1}^{A}w_{i}}\times 1,00,000.$$

Where $a_{i}$ represents the specific disease crude rate of the $i^{\text{th}}$ age group, and $w_{i}$ represents the reference standard for selection, and weight of the $i^{\text{th}}$ age group in the population.

The EAPC is a widely used metric in public health research to describe temporal trends in the epidemiological burden of disease. EAPC not only quantifies the rate of change in ASRs but also provides insights into potential future trends. It is calculated by fitting a regression line to the natural logarithm of the ASRs over time. The 95% confidence interval (CI) for the EAPC is derived from the linear regression model (26).


y=α+βx+ϵ

EAPC=100×(exp(β)−1)

In this analysis, y represents the natural logarithm of the ASR, and x represents the calendar year.

### 2.5 BAPC model projection

To project the future burden of breast cancer attributable to high red meat consumption, we employed the BAPC model, which is well-suited for handling complex, high-dimensional, and sparse data commonly encountered in large-scale epidemiological studies. This method addresses the non-identifiability problem inherent in traditional age-period-cohort (APC) models—caused by the linear dependency among age, period, and cohort effects—by introducing Bayesian priors. The model decomposes disease rates using a log-linear formulation:


$$\log(\lambda_{\text{apc}})=\mu +\alpha_{a}+\beta_{p}+\gamma_{c}$$

where $\lambda_{\text{apc}}$ is the disease rate for a given age-period-cohort stratum, μ represents the baseline effect, and $\alpha_{a}$, $\beta_{p}$ and $\gamma_{c}$ denote the additive effects of age, period, and cohort, respectively. For estimation, we utilized Integrated Nested Laplace Approximation (INLA) to fit the BAPC model. INLA combines Laplace approximation with modern numerical integration techniques under a Bayesian framework, offering a computationally efficient alternative to Markov Chain Monte Carlo (MCMC) methods, particularly for high-dimensional data with long computation times. To ensure model smoothness, second-order differences of all effects were assumed to follow independent zero-mean normal distributions.

The key assumptions of the BAPC model include separability of effects (i.e., age, period, and cohort impacts are independent and additive), smoothness constraints (minimal differences between adjacent groups), and data completeness (coverage across sufficient age and period ranges to reduce edge effects). The BAPC model demonstrates flexibility and robustness in analyzing time-series data and is especially suitable for long-term disease burden projections. Due to its adaptability and strong capacity for capturing temporal trends, the BAPC model has been widely applied in epidemiological research, particularly those involving complex cohort effects and structured population data (17, 27).

### 2.6 Statistical methods

All statistical analyses and data visualizations were performed using the World Health Organization’s Health Equity Assessment Toolkit and R software (version 4.3.3). To analyze the factors influencing the EAPC, Pearson correlation analysis was conducted to assess the correlations between the age-standardized high red meat diet index in 1990, the SDI in 2021, and the national-level EAPCs. The “BAPC” and “INLA” R packages were used to project disease burden trends from 2022 to 2030 based on the BAPC model.

For non-negative variables such as mortality counts, negative UIs indicate high uncertainty in areas with low counts or sparse data. In such cases, actual values should be interpreted as ≥0, with negative values reflecting model uncertainty under specific conditions rather than having biological significance (28). To improve the accuracy and interpretability of the results, all lower limits of UIs with negative values were truncated to 0, ensuring biological plausibility. The untruncated full range of UIs for the raw data can be found in the Supplementary materials. CIs are statistical tools used to describe the uncertainty range of estimates. The width of a CI reflects data variability and sample size; narrower CIs indicate more precise estimates, while wider CIs suggest greater uncertainty. In the EAPC analysis, the 95% CI indicates that 95% of intervals from repeated studies will contain the true value. If the EAPC value and its upper 95% CI are both <0, the true annual change rate of the ASR is significantly negative, indicating a statistically significant decline. Conversely, if the EAPC value and its lower 95% CI are both >0, the ASR is showing an upward trend. If the CI includes 0, it suggests that, based on the current data precision, we cannot rule out the possibility of no change (i.e., a zero annual change rate), indicating a stable trend. For trend analysis, *p*-values <0.05 were considered statistically significant.

## 3 Results

### 3.1 Global level

**Figure 1:**
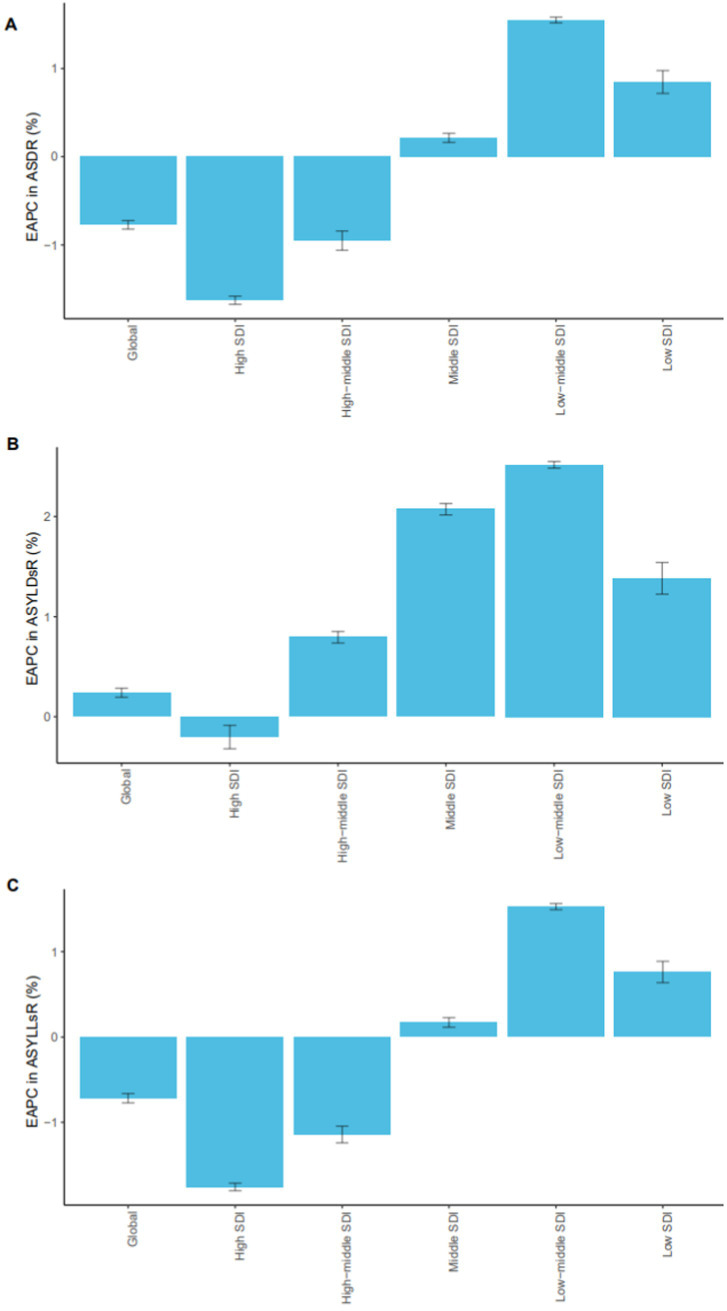
EAPC trends in breast cancer burden associated with high red meat diets by SDI regions (1990–2021). EAPCs in age-standardized mortality rates **(A)**, age-standardized YLD rates **(B)**, and age-standardized YLL rates **(C)** due to breast cancer associated to high red meat diets across different SDI regions from 1990 to 2021. The figure illustrates notable regional disparities, with higher EAPCs observed in lower SDI regions, indicating a growing disease burden in less developed areas. SDI, Sociodemographic Index; EAPC, Estimated Annual Percentage Change; YLLs, years of life lost; YLDs, years lived with disability.

In 2021, approximately 81,506 deaths globally were attributable to breast cancer related to high red meat consumption, representing an 80.83% increase compared to around 45,074 deaths in 1990. Despite this absolute increase, the global age-standardized mortality rate showed a declining trend, with an EAPC of −0.77 (95% CI: −0.82 to −0.72). Among the five SDI regions, the high SDI region exhibited a continued decline in age-standardized mortality rate (EAPC = −1.63, 95% CI: −1.67 to −1.58), YLL rate (EAPC = −1.76, 95% CI: −1.80 to −1.71), and YLD rate (EAPC = −0.20, 95% CI: −0.32 to −0.09), suggesting effective breast cancer prevention and control systems. In contrast, low, lower-middle, and upper-middle SDI regions showed an increasing trend across all indicators, with the upper-middle SDI region showing the most significant rise: age-standardized mortality rate (EAPC = 1.55, 95% CI: 1.52 to 1.58), YLL rate (EAPC = 1.53, 95% CI: 1.49 to 1.56), and YLD rate (EAPC = 2.52, 95% CI: 2.48 to 2.55) (Tables 1, 2; Figure 1; Supplementary Tables S1, S2).

**Table 1:** Trends in breast cancer deaths associated with high red meat diets, 1990–2021.

Characteristics	1990		2021		1990–2021
	Number (95% UI)	ASR (95% UI)	Number (95% UI)	ASR (95% UI)	EAPC (95% CI)
Global	45073.85(0 to 96427.94)	2.12 (0 to 4.53)	81506.23 (0 to 176183.52)	1.73 (0 to 3.74)	−0.77 (−0.82 to −0.72)
High SDI	19775.11 (0 to 42144.93)	3.32 (0 to 7.08)	23594.03 (0 to 50646.70)	2.07 (0 to 4.43)	−1.63 (−1.67 to −1.58)
High-middle SDI	12813.26 (0 to 27486.22)	2.39 (0 to 5.12)	20004.84 (0 to 43017.28)	1.88 (0 to 4.03)	−0.95 (−1.06 to −0.84)
Low SDI	1613.19 (0 to 3591.24)	1.20 (0 to 2.69)	4850.52 (0 to 10566.53)	1.58 (0 to 3.46)	0.85 (0.72 to 0.98)
Low-middle SDI	3087.26 (0 to 6804.99)	0.85 (0 to 1.87)	11451.25 (0 to 24931.22)	1.36 (0 to 2.96)	1.55 (1.52 to 1.58)
Middle SDI	7717.36 (0 to 16754.38)	1.28 (0 to 2.78)	21496.61 (0 to 46698.21)	1.42 (0 to 3.10)	0.21 (0.16 to 0.26)
Andean Latin America	179.57 (0 to 391.75)	1.48 (0 to 3.24)	549.24 (0 to 1253.12)	1.64 (0 to 3.75)	0.12 (0.00 to 0.24)
Australasia	449.43 (0 to 973.81)	3.59 (0 to 7.78)	597.38 (0 to 1297.49)	2.06 (0 to 4.47)	−1.85 (−1.92 to −1.79)
Caribbean	344.80 (0 to 743.98)	2.41 (0 to 5.20)	743.20 (0 to 1626.89)	2.50 (0 to 5.48)	0.22 (0.16 to 0.29)
Central Asia	703.11 (0 to 1497.43)	2.65 (0 to 5.64)	905.00 (0 to 1989.92)	1.92 (0 to 4.22)	−0.74 (−0.83 to −0.66)
Central Europe	2409.26 (0 to 5114.93)	3.02 (0 to 6.43)	3443.09 (0 to 7344.61)	2.87 (0 to 6.13)	−0.35 (−0.45 to −0.24)
Central Latin America	757.33 (0 to 1618.07)	1.54 (0 to 3.30)	2632.16 (0 to 5655.69)	1.87 (0 to 4.01)	0.48 (0.38 to 0.59)
Central Sub-Saharan Africa	186.61 (0 to 451.50)	1.37 (0 to 3.30)	564.21 (0 to 1367.94)	1.65 (0 to 3.99)	0.69 (0.41 to 0.98)
East Asia	5809.64 (0 to 12782.16)	1.15 (0 to 2.54)	13178.61 (0 to 29449.28)	1.11 (0 to 2.48)	−0.40 (−0.52 to-0.28)
Eastern Europe	4104.86 (0 to 8804.23)	2.72 (0 to 5.84)	4928.16 (0 to 10538.91)	2.60 (0 to 5.57)	−0.56 (−0.80 to −0.32)
Eastern Sub-Saharan Africa	791.22 (0 to 1783.09)	1.81 (0 to 4.07)	2503.27 (0 to 5589.09)	2.47 (0 to 5.51)	1.01 (0.91 to 1.11)
High-income Asia Pacific	1052.76 (0 to 2243.97)	0.93 (0 to 1.99)	2684.73 (0 to 5830.09)	1.18 (0 to 2.55)	0.77 (0.62 to 0.91)
High-income North America	7382.48 (0 to 15734.52)	3.92 (0 to 8.35)	8147.48 (0 to 17459.34)	2.31 (0 to 4.94)	−1.86 (−1.92 to −1.79)
North Africa and Middle East	914.52 (0 to 1988.20)	0.89 (0 to 1.92)	3901.04 (0 to 8462.31)	1.43 (0 to 3.09)	2.03 (1.79 to 2.26)
Oceania	43.16 (0 to 97.65)	2.36 (0 to 5.32)	127.00 (0 to 290.68)	2.62 (0 to 5.97)	0.34 (0.25 to 0.43)
South Asia	1897.72 (0 to 4241.89)	0.54 (0 to 1.21)	7157.56 (0 to 16140.44)	0.83 (0 to 1.88)	1.22 (1.08 to 1.36)
Southeast Asia	2125.17 (0 to 4724.12)	1.35 (0 to 3.00)	7646.64 (0 to 17229.32)	1.98 (0 to 4.47)	1.22 (1.16 to 1.28)
Southern Latin America	979.05 (0 to 2097.44)	3.91 (0 to 8.40)	1378.68 (0 to 3024.36)	2.88 (0 to 6.31)	−0.93 (−1.07 to −0.80)
Southern Sub-Saharan Africa	368.34 (0 to 827.25)	2.41 (0 to 5.43)	1153.66 (0 to 2545.84)	3.63 (0 to 8.01)	1.74 (1.48 to 1.99)
Tropical Latin America	1161.83 (0 to 2490.53)	2.22 (0 to 4.76)	3336.36 (0 to 7178.80)	2.33 (0 to 5.02)	−0.02 (−0.09 to 0.05)
Western Europe	12566.38 (0 to 26874.21)	4.12 (0 to 8.79)	12823.73 (0 to 27509.41)	2.45 (0 to 5.25)	−1.74 (−1.79 to −1.68)
Western Sub-Saharan Africa	846.62 (0 to 1806.86)	1.68 (0 to 3.58)	3105.038 (0 to 6982.94)	2.62 (0 to 5.88)	1.59 (1.45 to 1.73)

ASR, age-standardized rate; EAPC, estimated annual percentage change; CI, confidence interval; UI, uncertainty interval; SDI, socio-demographic index. The lower bound of negative UIs has been truncated to 0; the full range of the raw data can be found in Supplementary Table S1.

**Table 2:** YLLs and YLDs trends for breast cancer associated with high red meat diets, 1990–2021.

Characteristics	YLLs			YLDs		
	2021		1990–2021	2021		1990–2021
	Number (95% UI)	ASR (95% UI)	EAPC (95% CI)	Number (95% UI)	ASR (95% UI)	EAPC (95% CI)
Global	2263019.99 (0 to 4893977.19)	47.42 (0 to 102.56)	−0.72 (−0.77 to −0.66)	188698.65 (0 to 447393.84)	3.95 (0 to 9.37)	0.24 (0.19 to 0.28)
High SDI	519470.73 (0 to 1110993.13)	52.41 (0 to 112.05)	−1.76 (−1.80 to −1.71)	75768.82 (0 to 179867.42)	7.48 (0 to 17.81)	−0.20 (−0.32 to-0.09)
High-middle SDI	523993.56 (0 to 1128203.86)	50.09 (0 to 107.93)	−1.14 (−1.24 to −1.05)	49505.29 (0 to 117071.11)	4.71 (0 to 11.13)	0.79 (0.74 to 0.85)
Low SDI	167947.84 (0 to 365313.67)	46.83 (0 to 101.98)	0.76 (0.64 to 0.89)	4804.37 (0 to 11394.83)	1.37 (0 to 3.25)	1.38 (1.23 to 1.54)
Low-middle SDI	382183.33 (0 to 832430.28)	41.81 (0 to 91.08)	1.53 (1.49 to 1.56)	14722.64 (0 to 34850.96)	1.63 (0 to 3.87)	2.52 (2.48 to 2.55)
Middle SDI	666615.45 (0 to 1447540.73)	42.40 (0 to 92.15)	0.17 (0.12 to 0.23)	43703.89 (0 to 102517.12)	2.79 (0 to 6.53)	2.07 (2.02 to 2.13)
Andean Latin America	16475.75 (0 to 37577.14)	47.58 (0 to 108.51)	−0.03 (−0.15 to 0.08)	860.97 (0 to 2118.04)	2.50 (0 to 6.14)	1.88 (1.75 to 2.00)
Australasia	13397.75 (0 to 28927.68)	52.42 (0 to 113.00)	−2.06 (−2.12 to −2.00)	2104.17 (0 to 4967.84)	8.07 (0 to 19.11)	−0.01 (−0.18 to 0.15)
Caribbean	20586.69 (0 to 45332.69)	70.24 (0 to 154.76)	0.13 (0.08 to 0.18)	1208.27 (0 to 2872.49)	4.11 (0 to 9.77)	0.87 (0.77 to 0.96)
Central Asia	28436.83 (0 to 62474.10)	55.36 (0 to 121.69)	−1.04 (−1.12 to −0.95)	1408.75 (0 to 3329.70)	2.79 (0 to 6.58)	−0.20 (−0.27 to −0.13)
Central Europe	77248.61 (0 to 165024.48)	70.70 (0 to 151.35)	−0.71 (−0.82 to −0.6)	6022.84 (0 to 14138.37)	5.53 (0 to 13.03)	0.96 (0.81 to 1.11)
Central Latin America	79648.47 (0 to 170577.62)	54.78 (0 to 117.38)	0.45 (0.35 to 0.55)	6242.93 (0 to 14740.22)	4.30 (0 to 10.15)	2.00 (1.90 to 2.10)
Central Sub-Saharan Africa	19833.93 (0 to 48218.66)	48.31 (0 to 117.14)	0.57 (0.28 to 0.86)	547.68 (0 to 1396.23)	1.39 (0 to 3.50)	1.22 (0.86 to 1.57)
East Asia	395307.81 (0 to 888101.54)	33.3 (0 to 74.92)	−0.47 (−0.58 to −0.36)	40206.48 (0 to 95775.39)	3.37 (0 to 8.04)	2.68 (2.59 to 2.77)
Eastern Europe	125962.24 (0 to 269340.76)	70.13 (0 to 150.03)	−0.96 (−1.21 to −0.72)	9220.61 (0 to 21540.73)	5.05 (0 to 11.82)	0.48 (0.37 to 0.59)
Eastern Sub-Saharan Africa	86067.48 (0 to 192910.77)	69.69 (0 to 155.67)	0.84 (0.74 to 0.94)	2473.04 (0 to 5793.23)	2.07 (0 to 4.82)	1.52 (1.39 to 1.66)
High-income Asia Pacific	62214.66 (0 to 133956.64)	34.23 (0 to 73.41)	0.34 (0.17 to 0.52)	10435.18 (0 to 25006.91)	5.46 (0 to 13.12)	1.95 (1.77 to 2.14)
High-income North America	184280.44 (0 to 393552.62)	58.24 (0 to 124.41)	−2.07 (−2.15 to −1.99)	30818.34 (0 to 72398.15)	9.40 (0 to 22.15)	−1.00 (−1.11 to −0.89)
North Africa and Middle East	129437.19 (0 to 280645.43)	41.92 (0 to 90.89)	1.70 (1.49 to 1.90)	11330.21 (0 to 27106.34)	3.79 (0 to 9.04)	3.73 (3.48 to 3.98)
Oceania	4642.40 (0 to 10698.20)	82.25 (0 to 188.48)	0.37 (0.28 to 0.46)	128.22 (0 to 308.23)	2.37 (0 to 5.67)	0.17 (0.05 to 0.30)
South Asia	238777.50 (0 to 537235.48)	25.68 (0 to 57.84)	1.21 (1.06 to 1.35)	8161.27 (0 to 19667.19)	0.89 (0 to 2.14)	2.21 (2.02 to 2.40)
Southeast Asia	251535.17 (0 to 567905.67)	60.88 (0 to 137.3)	1.08 (1.02 to 1.14)	10606.47 (0 to 25752.2)	2.59 (0 to 6.30)	2.30 (2.25 to 2.34)
Southern Latin America	33760.64 (0 to 73599.89)	73.63 (0 to 160.39)	−1.09 (−1.19 to −0.99)	2204.28 (0 to 5172.60)	4.77 (0 to 11.22)	0.36 (0.23 to 0.50)
Southern Sub-Saharan Africa	35385.65 (0 to 78321.66)	97.30 (0 to 214.99)	1.72 (1.43 to 2.02)	1249.36 (0 to 2948.50)	3.49 (0 to 8.19)	2.20 (1.98 to 2.42)
Tropical Latin America	98484.27 (0 to 211614.00)	67.54 (0 to 145.15)	−0.06 (−0.13 to 0.01)	5010.17 (0 to 11811.43)	3.44 (0 to 8.12)	1.19 (1.09 to 1.28)
Western Europe	257974.08 (0 to 551331.84)	59.57 (0 to 127.12)	−2.01 (−2.06 to −1.95)	35413.55 (0 to 84908.17)	8.21 (0 to 19.75)	0.01 (−0.16 to 0.18)
Western Sub-Saharan Africa	103562.42 (0 to 233422.03)	74.46 (0 to 167.52)	1.56 (1.41 to 1.71)	3045.85 (0 to 7238.35)	2.25 (0 to 5.33)	2.20 (2.03 to 2.38)

ASR, age-standardized rate; EAPC, estimated annual percentage change; CI, confidence interval; UI, uncertainty interval; SDI, socio-demographic index; YLLs, years of life lost; YLDs, years lived with disability. The lower bound of negative UIs has been truncated to 0; the full range of the raw data can be found in Supplementary Table S2.

In women, the number of deaths increased from approximately 44,492 in 1990 to around 79,957 in 2021, while the age-standardized mortality rate decreased from 3.83 (95% UI: 0 to 8.19) to 3.18 (95% UI: 0 to 6.88), reflecting the overall population trend. In high SDI and upper-middle SDI regions, age-standardized mortality rates and YLL rates in women decreased over time, reflecting the benefits of early detection, timely treatment, and advanced therapeutic strategies. In contrast, an increasing trend was observed in low, lower-middle, and middle SDI regions, which may reflect challenges related to limited healthcare resources.

In men, the age-standardized mortality rate slightly increased from 0.06 (95% UI: 0 to 0.13) to 0.07 (95% UI: 0 to 0.16), with minimal variation across SDI regions. The overall trend remained stable. However, men in low SDI regions had significantly higher age-standardized mortality and YLL rates compared to those in high SDI regions. The age-standardized YLL rate in low SDI regions (4.02 per 100,000) was nearly four times higher than that in high SDI regions (1.04 per 100,000), suggesting disparities in treatment accessibility (Supplementary Figure S1).

### 3.2 Regional level

In 2021, the highest age-standardized mortality rates related to breast cancer attributable to high red meat consumption were observed in Southern Sub-Saharan Africa (3.63, 95% UI: 0 to 8.01), Southern Latin America (2.88, 95% UI: 0 to 6.31), and Central Europe (2.87, 95% UI: 0 to 6.13). In contrast, the lowest rates were seen in South Asia (0.83, 95% UI: 0 to 1.88), East Asia (1.11, 95% UI: 0 to 2.48), and High-income Asia Pacific (1.18, 95% UI: 0 to 2.55). The YLL rates followed a similar geographical distribution, with Southern Sub-Saharan Africa reaching 97.30 per 100,000 (95% UI: 0 to 214.99). However, the YLD rates displayed a different geographic pattern, with High-income North America (9.40, 95% UI: 0 to 22.15), Western Europe (8.21, 95% UI: 0 to 19.75), and Australasia (8.07, 95% UI: 0 to 19.11) identified as the major burden hotspots.

From 1990 to 2021, significant regional heterogeneity was observed in the mortality trends. North Africa and the Middle East (EAPC = 2.03, 95% CI: 1.79 to 2.26), Southern Sub-Saharan Africa (EAPC = 1.74, 95% CI: 1.48 to 1.99), and Western Sub-Saharan Africa (EAPC = 1.59, 95% CI: 1.45 to 1.73) showed the fastest increases in mortality, likely due to a surge in red meat consumption and low screening coverage. In contrast, High-income North America (EAPC = −1.86, 95% CI: −1.92 to −1.79), Australasia (EAPC = −1.85, 95% CI: −1.92 to −1.79), and Western Europe (EAPC = −1.74, 95% CI: −1.79 to −1.68) experienced the largest decreases in mortality.

Age-standardized YLL rates increased in 11 regions, with the most significant increases observed in Southern Sub-Saharan Africa (EAPC = 1.72, 95% CI: 1.43 to 2.02), North Africa and the Middle East (EAPC = 1.70, 95% CI: 1.49 to 1.90), and Western Sub-Saharan Africa (EAPC = 1.56, 95% CI: 1.41 to 1.71). In contrast, High-income North America (EAPC = −2.07, 95% CI: −2.15 to −1.99), Australasia (EAPC = −2.06, 95% CI: −2.12 to −2.00), and Western Europe (EAPC = −2.01, 95% CI: −2.06 to −1.95) saw the largest decreases. While 85.7% of regions showed a general increase in YLD rates, particularly North Africa and the Middle East (EAPC = 3.73, 95% CI: 3.48 to 3.98), High-income North America saw the most significant decline (EAPC = −1.00, 95% CI: −1.11 to −0.89).

**Figure 2:**
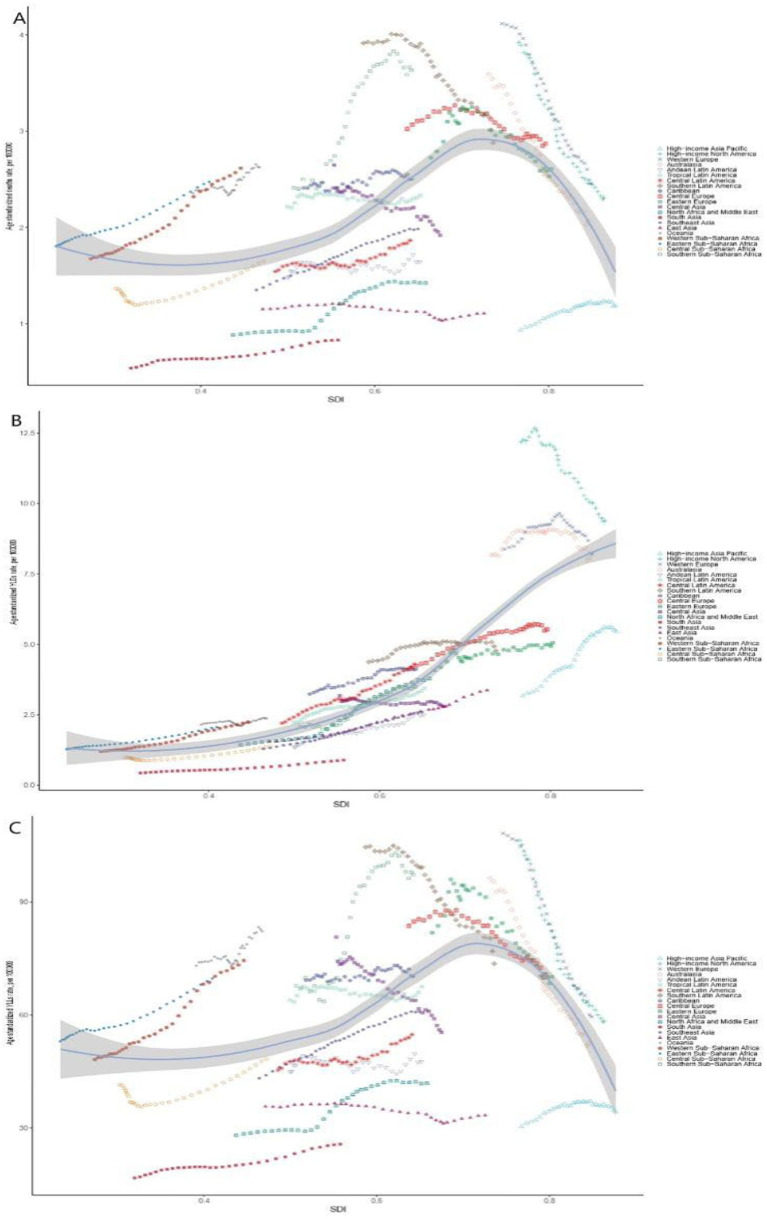
Trends of breast cancer burden associated with high red meat diets in SDI and regional groups in 2021. Age-standardized mortality rates **(A)**, age-standardized YLDs rates **(B)**, and age-standardized YLLs rates **(C)** associated with breast cancer and high red meat diets across different SDI and GBD regional groups in 2021. Notably, high SDI regions still exhibited a relatively high burden of disease. SDI, Sociodemographic Index; YLLs, years of life lost; YLDs, years lived with disability.

In 2021, SDI regions showed a positive correlation with age-standardized mortality rates, YLL rates, and YLD rates (*r* = 0.4109, *r* = 0.3344, *r* = 0.905; all *p* < 0.01), indicating that regions with higher socioeconomic development have a higher disease burden. From 1990 to 2021, countries such as Oceania and High-income North America experienced a higher-than-expected breast cancer disease burden related to high red meat consumption, while regions like Central Sub-Saharan Africa, South Asia, Andean Latin America, Southeast Asia, and East Asia remained below expectations throughout the period (Tables 1, 2; Figure 2; Supplementary Tables S1, S2).

In men, Eastern Sub-Saharan Africa observed the highest age-standardized mortality rate, YLL rate, and YLD rate. In women, Southern Sub-Saharan Africa had the highest age-standardized mortality rate and YLL rate, while High-income North America had the highest YLD rate. South Asia exhibited the lowest age-standardized mortality rate, YLL rate, and YLD rate (Supplementary Figure S2).

### 3.3 Country level

In 2021, the highest age-standardized mortality rate (ASMR) and YLL rate were observed in the Republic of Croatia (5.15 per 100,000 and 152.07 per 100,000, respectively). Conversely, the countries with the lowest disease burden were the Republic of Honduras (0.53 per 100,000 and 32.09 per 100,000), Bosnia and Herzegovina (0.42 per 100,000 and 16.13 per 100,000), and the People’s Republic of China (0.55 per 100,000 and 15.74 per 100,000).

From 1990 to 2021, the age-standardized YLL rate increased in 128 countries. The largest increases were observed in Taiwan (Province of China) (EAPC = 3.60, 95% CI: 2.84 to 4.35), Zimbabwe (EAPC = 3.34, 95% CI: 2.52 to 4.16), and Niue (EAPC = 3.04, 95% CI: 2.58 to 3.50), while the most significant declines were recorded in the Grand Duchy of Luxembourg (EAPC = −2.96, 95% CI: −3.07 to −2.85), Tuvalu (EAPC = −2.57, 95% CI: −2.65 to −2.49), and the Republic of Haiti (EAPC = −2.56, 95% CI: −2.74 to −2.38).

Additionally, 86.8% of countries showed an increase in age-standardized YLD rates, with the largest increases observed in Taiwan (Province of China) (EAPC = 6.30, 95% CI: 5.45 to 7.16), the People’s Republic of China (EAPC = 4.30, 95% CI: 3.49 to 5.12), and the Hashemite Kingdom of Jordan (EAPC = 4.04, 95% CI: 3.65 to 4.42). The Czech Republic exhibited the most notable decrease (EAPC = −1.23, 95% CI: −1.64 to −0.82) (Supplementary Tables S1, S2).

**Figure 3:**
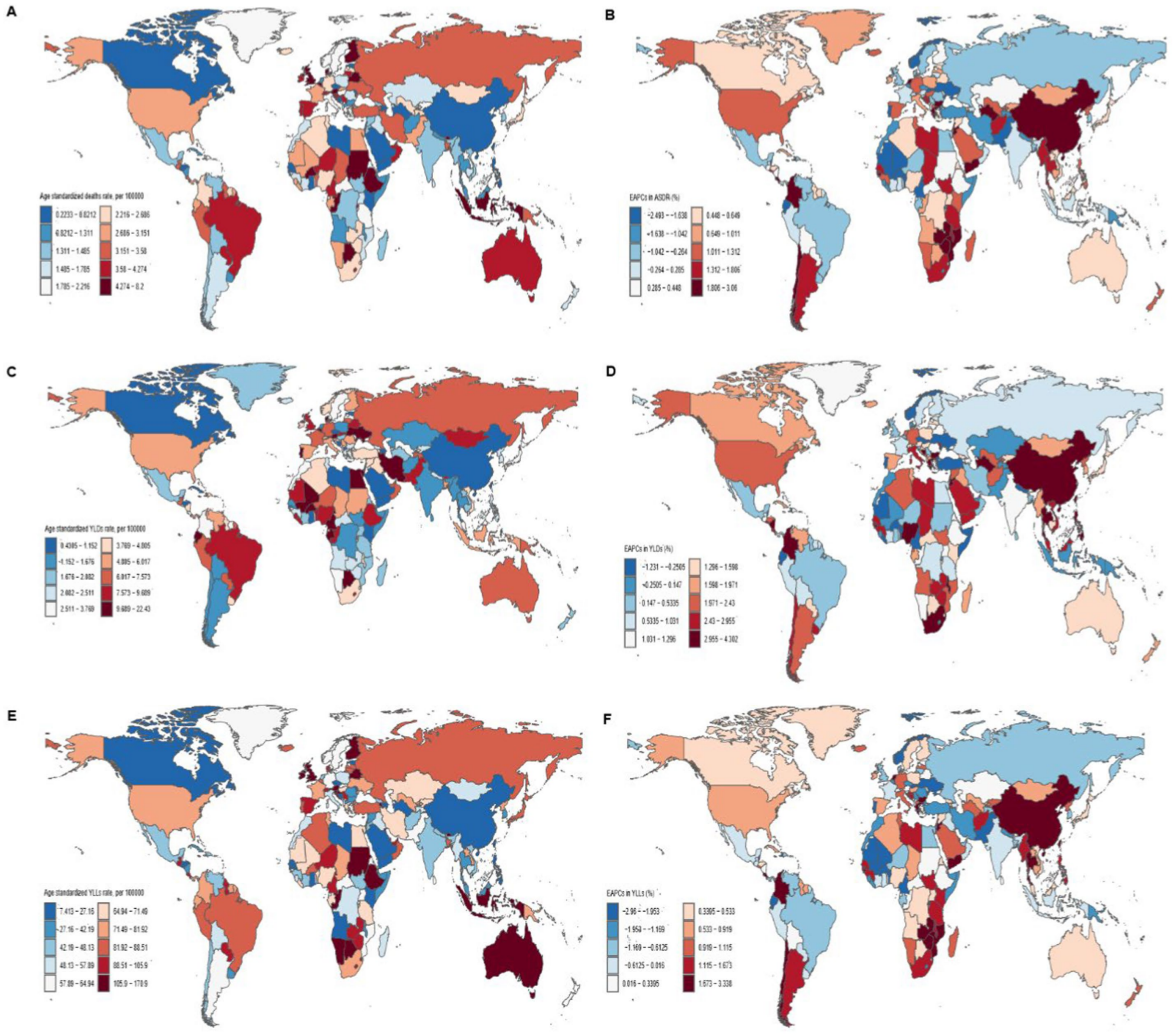
Global distribution and trends in breast cancer burden related to high red meat diets in 204 countries. Maps illustrate the age-standardized mortality rates **(A)**, age-standardized YLDs rates **(C)**, and age-standardized YLLs rates **(E)** from breast cancer related to high red meat diets across 204 countries in 2021. EAPCs in age-standardized mortality rates **(B)**, YLDs **(D)**, and YLLs **(F)** from breast cancer associated with high red meat diet in 204 countries from 1990 to 2021. There are persistent differences in disease burden and growth trends between countries, with notable variations across different SDI regions. EAPC, Estimated Annual Percentage Change; YLLs, years of life lost; YLDs, years lived with disability.

In 2021, a positive correlation was found between SDI levels and age-standardized mortality, YLL, and YLD rates (*r* = 0.1815, *r* = 0.0494, *r* = 0.0494; *p* < 0.001), indicating that countries with higher socioeconomic development experienced a greater disease burden from breast cancer attributable to high red meat consumption. Countries such as Ethiopia, Australia, Sudan, and the United Kingdom of Great Britain and Northern Ireland had higher-than-expected disease burdens, while countries such as Senegal, Saudi Arabia, China, and Norway had lower-than-expected burdens (Figure 3; Supplementary Figure S3).

### 3.4 Global burden of breast cancer attributable to diets high in red meat by 2030 projected

**Figure 4:**
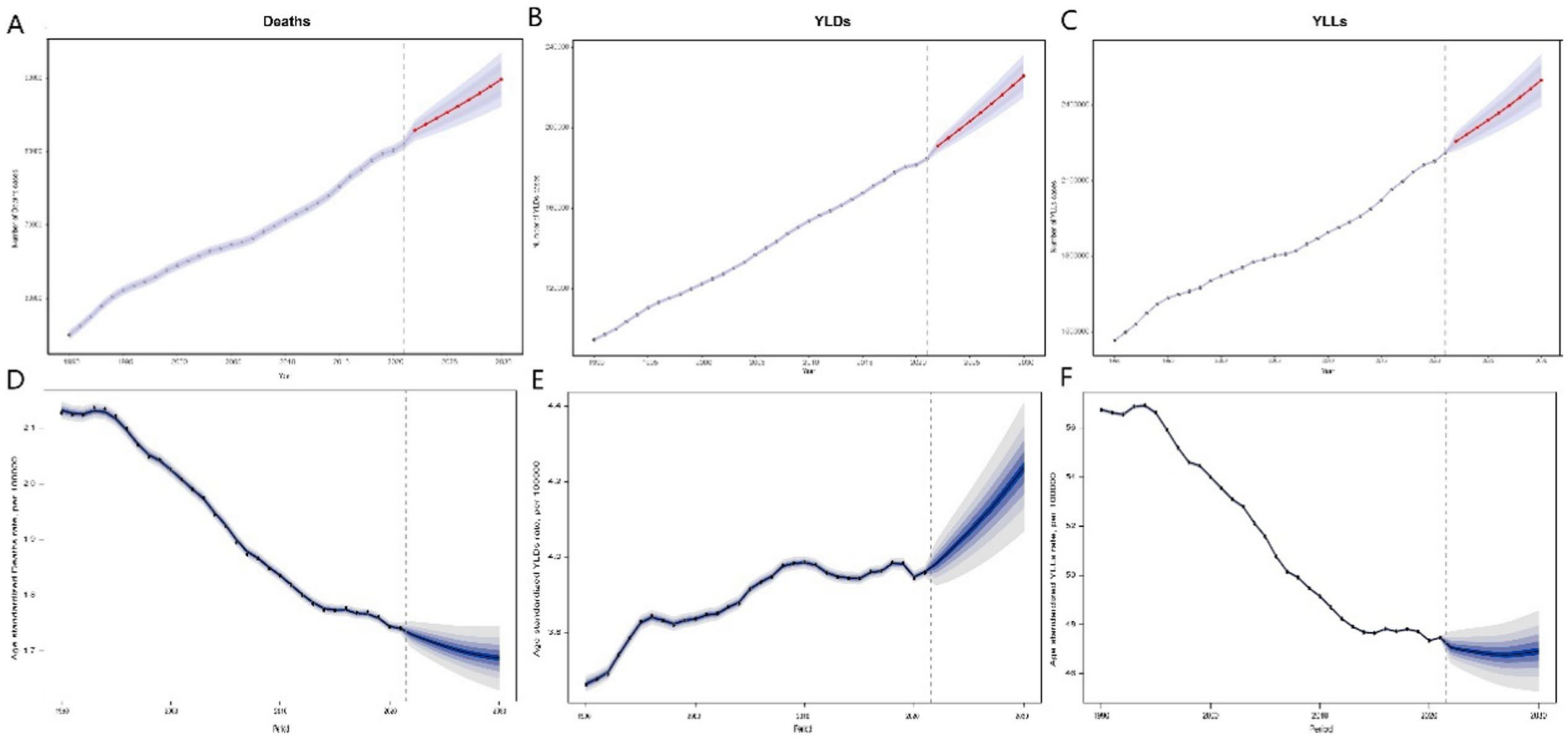
Predicted trends in deaths, YLDs, and YLLs from breast cancer associated with high red meat diets from 2022 to 2030. Predicted deaths **(A)**, YLDs **(B)**, and YLLs **(C)** from 2022 to 2030. Error bars represent the 95% confidence intervals of the predicted values. Predicted age-standardized mortality rates **(D)**, age-standardized YLDs rate **(E)**, and age-standardized YLLs rate **(F)** from 2020 to 2030, with hollow points representing observed values and shaded areas indicating the predicted distribution between the 2.5 and 97.5% percentiles. The predicted mean values are shown as solid lines. Vertical dashed lines represent the start of the prediction period. This figure illustrates the forecasted trends in breast cancer burden associated with high red meat consumption, with deaths and YLDs projected to increase, while age-standardized mortality rates and YLLs are predicted to show a decreasing trend, reflecting a decline in premature mortality but a rise in disability due to the disease. YLLs, years of life lost; YLDs, years lived with disability.

Using the BAPC model, we projected the global burden of breast cancer attributable to high red meat consumption from 2022 to 2030. The number of related deaths is expected to continue rising, reaching approximately 89,826.50 cases by 2030 (95% UI: 86,138.15 to 93,514.85). The age-standardized mortality rate is projected to decrease, reaching an estimated 1.69 (95% UI: 1.62 to 1.76) by 2030. Meanwhile, the age-standardized YLL rate is also expected to decline, ultimately reaching 46.91 (95% UI: 45.0 to 48.87), which is below the historical minimum. However, the age-standardized YLD rate is projected to increase significantly, reaching a new historical peak of 4.24 (95% UI: 4.34 to 4.44; Figure 4).

## 4 Discussion

This study highlights the significant impact of high red meat diets on the burden of breast cancer, particularly in the context of global dietary transitions and social development. The burden varies markedly across regions and SDI levels. In 2021, approximately 80,000 breast cancer deaths worldwide were attributable to high red meat diets, representing an increase of about 81% compared to the number of deaths in 1990. Over the past three decades, age-standardized mortality rates and YLL rates have declined, while the absolute number of deaths and age-standardized YLD rates have increased. These trends suggest that increased red meat consumption remains a substantial global health burden for breast cancer, with the overall burden potentially continuing to rise. By analyzing trends at the global, regional, and national levels, this study reveals significant disparities in disease burden across countries, regions, genders, and socioeconomic strata, underscoring the urgent need for targeted public health strategies.

The impact of high red meat diets on the burden of breast cancer varies significantly across regions worldwide. A previous summary of data from 1992 to 2013 revealed that red meat consumption in high-income countries was approximately five times higher than that in low-income countries (29). Red meat contains substantial amounts of heme iron, which has potential pro-oxidative properties that may induce oxidative stress and DNA damage, thereby potentially facilitating the initiation and progression of breast tumors (30, 31). In addition, the residues of exogenous hormones in red meat—stemming from the use of antibiotics and growth hormones in cattle and sheep farming—may increase the risk of breast cancer through endocrine-disrupting mechanisms (32, 33). As red meat consumption has traditionally been higher in affluent countries, these biological mechanisms may partly explain the elevated prevalence of breast cancer in these regions. Although analyses of health disparities show that the burden of breast cancer is more geographically concentrated in high-income countries, recent years have witnessed a decline in red meat consumption in these areas, partly attributable to the dissemination of dietary guidelines and the rising awareness of public health (34). Moreover, high-income countries have widely implemented breast cancer screening programs and continuously optimized treatment strategies, which have partially mitigated the disease burden associated with high red meat diets. However, it is noteworthy that although overall red meat intake has declined in these countries, the disease burden related to high red meat diets has not decreased correspondingly. This discrepancy may be closely linked to the modernization of lifestyles, the westernization of dietary patterns, and the insufficient implementation of dietary interventions among certain high-risk populations. For example, red meat is considered a key component of traditional diets, and many individuals have not effectively reduced their intake. Moreover, certain populations continue to prepare red meat using high-temperature methods such as grilling and frying. Cooking red meat at high temperatures generates carcinogenic compounds—such as heterocyclic amines (HCAs) and polycyclic aromatic hydrocarbons (PAHs). These compounds have been shown to be significantly associated with the development of breast cancer by disrupting DNA replication and repair processes and inducing genetic mutations (35–38). First, high-income countries can implement comprehensive interventions across multiple levels, including the continuous optimization of disease screening programs and the integration of dietary intervention guidelines to reduce red meat consumption, with the goal of keeping the maximum daily intake of red meat within a reasonable proportion of total dietary intake (6). Second, the promotion of healthy eating concepts can be carried out through various media channels, along with the strengthening of nutrition education in schools and communities. Meanwhile, multiple studies have also confirmed that natural compounds can reduce the formation of carcinogens during the lipid peroxidation of meat. For instance, phenolic compounds in olives can rapidly reduce heme-induced lipid peroxidation and scavenge free radicals such as 1,1-diphenyl-2-picrylhydrazyl (DPPH). Vitamin E has been shown to inhibit iron-induced damage, while vitamin C can reduce free iron to ferrous iron, thereby stimulating the initiation and propagation of free radical reactions (39, 40). Therefore, it is recommended to use plant extracts instead of synthetic antioxidants prior to the intensive cooking of meat products.

In middle-SDI regions, such as Eastern Europe, Latin America, and parts of Asia, the burden of breast cancer is also gradually increasing. With economic development, residents in these areas are more inclined to purchase affordable but highly processed meat products, which are often rich in additives, hormone residues, and saturated fats. These characteristics not only alter the nutritional quality of the food but may also contribute to an increased production of carcinogenic metabolites by disrupting the gut microbiota environment (41, 42). For these regions, implementing a “health tax” or consumption surcharge on processed meat products could be considered to increase their retail prices and curb excessive consumption. At the same time, subsidies could be provided for unprocessed lean meats and plant-based protein sources, such as legumes and nuts, to reduce the cost for consumers (6). Food companies should be encouraged to reduce the use of saturated fats, additives, and hormones in processed meat products, and to promote healthier curing techniques and the use of natural antioxidants. Through technological innovation and strengthened regulation, promoting greener and healthier models of meat production can help meet global protein demands while reducing diet-related health risks. Moreover, poultry consumption, as compared to red meat, may be associated with lower levels of mutagenic activity, oxidative stress, and DNA damage (10). At the same time, enhancing public awareness and promoting healthy dietary patterns remain essential components of comprehensive health strategies.

In low-SDI regions, the disease burden has increased most significantly in recent years. Due to limited medical resources, inadequate diagnostic capacity, and lagging health education, breast cancer is often diagnosed at an advanced stage. Although plant-based diets have traditionally been predominant in these regions, red meat consumption has risen markedly with economic growth and the influence of Western dietary culture—particularly among urban populations. A type of animal sugar molecule, N-glycolylneuraminate (Neu5Gc), which is abundant in red meat, has been shown to accumulate in human tissues and induce a chronic inflammatory state (43). Chronic inflammation has been widely recognized as one of the promoting factors for breast cancer, suggesting that the health risks associated with red meat consumption are not limited to chemical carcinogens but also involve immune-inflammatory mechanisms. It is therefore recommended to promote an “anti-inflammatory diet”—increasing the intake of whole grains, fruits, vegetables, and tea, while reducing the consumption of processed meats and high-fat foods—to suppress chronic inflammation, lower the risk of breast cancer, and potentially improve overall survival among breast cancer survivors (44). In addition, research has shown that individuals with genetic susceptibility—such as those carrying null genotypes of GSTM1 and GSTT1—have reduced detoxification capacity and are more likely to experience DNA damage after exposure to carcinogens like heterocyclic amines (HCAs), leading to a significantly increased risk of breast cancer (45). Notably, there are significant ethnic differences in the prevalence of these gene polymorphisms, with higher frequencies of such null genotypes observed among Asian and Caucasian populations. This suggests that genetic background may exacerbate the health risks faced by populations in low-income countries (46). In these regions, it is essential to introduce cost-effective and easy-to-implement early screening techniques into primary healthcare systems, such as Clinical Breast Examination and rural ultrasound screening. Additionally, public education campaigns on high-risk signs of breast cancer and dietary risk factors should be conducted to promote the health concepts of “early detection, early diagnosis, and early treatment” as well as “balanced diet and limited red meat consumption” among residents in remote and resource-limited areas. These efforts aim to enhance healthcare-seeking behavior and facilitate positive dietary changes.

At the national level, countries with the highest age-standardized mortality and YLL rates—such as Indonesia, Croatia, and nations in sub-Saharan Southern Africa—reflect the geographical heterogeneity of disease burden. These disparities are influenced not only by the trajectories of nutritional transition and economic development but also by deep-rooted cultural, religious, and geographic factors. For example, coastal countries such as Japan and Norway traditionally maintain fish-based dietary patterns, with relatively low consumption of red meat (47). In India, religious restrictions limit beef consumption, and in some Southeast Asian countries with Buddhist traditions, dietary protein is primarily derived from grains, fish, legumes, and dairy products rather than red meat (48). This dietary pattern contributes to relatively lower breast cancer incidence rates in certain regions. In contrast, inland countries with no prominent religious dietary restrictions, such as certain Latin American and Eastern European nations, exhibit a more widespread presence of red meat-related risk factors. Therefore, it is recommended to encourage a reduction in the consumption of processed red meat and promote plant-based and low-saturated fat protein sources, such as legumes, nuts, and fish. At the same time, the preference for red meat among certain cultural groups is closely tied to its symbolic significance in festive meals and social customs, making dietary adjustments more challenging. In response to this, it is advisable to advocate for the inclusion of fish and soy-based products in festive and social occasions, thereby incorporating healthy eating principles into traditional practices.

The disease burden projections from 2022 to 2030 indicate that, despite the continued increase in breast cancer burden related to high red meat diets due to population growth and aging, the age-standardized burden may slightly decrease over time. This could reflect global efforts in cancer prevention, dietary interventions, and improvements in healthcare accessibility. However, the decline is expected to be modest, and regional disparities are likely to persist, highlighting the need for targeted policy strategies and public health initiatives, particularly in low and middle SDI countries.

High red meat diets negatively impact breast tissue through multiple mechanisms, including oxidative stress, genetic mutations, endocrine disruption, chronic inflammation, and gut microbiome imbalance. And on a global scale, it jointly shapes the geographical landscape of the breast cancer disease burden through socioeconomic status, cultural customs, and genetic differences. Therefore, it is particularly urgent and necessary to develop region-specific intervention policies and implement targeted prevention strategies based on molecular mechanisms and population characteristics.

The primary strength of this study lies in its use of globally comprehensive disease burden data, combined with the BAPC model to project trends in breast cancer burden associated with high red meat consumption. This approach demonstrates high spatiotemporal sensitivity and predictive accuracy, offering solid scientific evidence for policymakers. However, the attribution analysis has the following potential confounding factors: Firstly, dietary data mainly rely on the food balance sheet at the national level, making it difficult to fully reflect the differences in individual actual intake or cooking methods (such as pickling, high-temperature grilling). Secondly, although the model incorporated covariates such as SDI and smoking rates, it did not fully cover potential confusions such as genetic susceptibility (such as dietary interactions in BRCA1 mutation carriers) or exposure to environmental pollutants (such as hormone residues in meat). These residual confusions may affect the accuracy of PAF estimation. Although the GBD database provides extensive global health data, in low SDI regions, due to limited medical resources and inadequate health information systems, breast cancer-related mortality data may suffer from underreporting or misclassification. Additionally, there are significant differences in dietary survey coverage, methodologies, and data quality across different countries and regions, further increasing the uncertainty of the estimates. Therefore, the results of this study should be regarded as statistical associations rather than definitive causal evidence. Future research should focus on improving data collection, individual exposure assessment, and model design, and validate such analyses in more populations and regions to enhance the accuracy and reliability of the estimates. At the same time, the development of innovative research methods, such as genome-wide association studies and multi-omics analyses, can more comprehensively reveal the biological basis of this complex relationship.

## 5 Conclusion

In summary, the burden of breast cancer associated with high red meats diets shows significant global differences. High red meat diets have become one of the key drivers of the increasing burden of breast cancer, particularly in low and middle SDI regions, where it poses an even greater threat to public health. Through comprehensive dietary interventions, optimization of healthcare resource allocation, and international collaboration, this disease burden can be effectively reduced, contributing to further advancements in global breast cancer prevention and control efforts.

## Data Availability

The original contributions presented in the study are included in the article/Supplementary material, further inquiries can be directed to the corresponding author.

## Footnotes

^1^ http://ghdx.healthdata.org/gbd-2021/datainput-sources
