# Supplementary material for: Global burden of disease changes related to high red meat diets and breast cancer from 1990 to 2021 and its prediction up to 2030
Source: Front Nutr. 2025 Jun 4;12:1586299. doi: 10.3389/fnut.2025.1586299 (PMC12173928; doi:10.3389/fnut.2025.1586299)
Supplement: Supplementary file 1 [file Table_1.docx]

Supplementary Material

# Supplementary Tables

**Table S1. Trends in Breast Cancer Deaths Associated with High Red Meat Diets 1990–2021.**

| **Characteristics** | **1990** | | **2021** | |
| --- | --- | --- | --- | --- |
|  | **Number (95% UI)** | **ASR (95% UI)** | **Number (95% UI)** | **ASR (95% UI)** |
| Global | 45073.85(-14.44 to 96427.94) | 2.12 (-0.00 to 4.53) | 81506.23 (-26.80 to 176183.52 ) | 1.73 (-0.00 to 3.74) |
| Sociodemographic index |  |  |  |  |
| Low SDI | 1613.19 (-0.32 to 3591.24) | 1.20 (-0.00 to 2.69) | 4850.52 (-0.98 to 10566.53) | 1.58 (-0.00 to 3.46) |
| Low-middle SDI | 3087.26 (-0.63 to 6804.99) | 0.85 (-0.00 to 1.87) | 11451.25 (-3.24 to 24931.22 ) | 1.36 (-0.00 to 2.96) |
| Middle SDI | 7717.36 (-2.30 to 16754.38) | 1.28 (-0.00 to 2.78) | 21496.61 (-6.89 to 46698.21) | 1.42 (-0.00 to 3.10) |
| High-middle SDI | 12813.26 (-4.96 to 27486.22) | 2.39 (-0.00 to 5.12) | 20004.84 (-8.20 to 43017.28) | 1.88 (-0.00 to 4.03) |
| High SDI | 19775.11 (-7.69 to 42144.93) | 3.32 (-0.00 to 7.08) | 23594.03 (-10.25 to 50646.70) | 2.07 (-0.00 to 4.43) |
| GBD regions |  |  |  |  |
| Andean Latin America | 179.57 (-0.07 to 391.75) | 1.48 (-0.00 to 3.24 ) | 549.24 (-0.27 to 1253.12) | 1.64 (-0.00 to 3.75) |
| Australasia | 449.43 (-0.05 to 973.81) | 3.59 (-0.00 to 7.78) | 597.38 (-0.15 to 1297.49) | 2.06 (-0.00 to 4.47) |
| Caribbean | 344.80 (-0.10 to 743.98) | 2.41 (-0.00 to 5.20) | 743.20 (-0.21 to 1626.89) | 2.50 (-0.00 to 5.48) |
| Central Asia | 703.11 (-0.34 to 1497.43) | 2.65 (-0.00 to 5.64) | 905.00 (-0.58 to 1989.92) | 1.92 (-0.00 to 4.22) |
| Central Europe | 2409.26 (-1.03 to 5114.93) | 3.02 (-0.00 to 6.43) | 3443.09 (-1.68 to 7344.61) | 2.87 (-0.00 to 6.13) |
| Central Latin America | 757.33 (-0.27 to 1618.07) | 1.54 (-0.00 to 3.30) | 2632.16 (-1.30 to 5655.69) | 1.87 (-0.00 to 4.01) |
| Central Sub-Saharan Africa | 186.61 (-0.043 to 451.50) | 1.37 (-0.00 to 3.30) | 564.21 (-0.12 to 1367.94) | 1.65 (-0.00 to 3.99) |
| East Asia | 5809.64 (-2.36 to 12782.16) | 1.15 (-0.00 to 2.54) | 13178.61 (-5.29 to 29449.28) | 1.11 (-0.00 to 2.48) |
| Eastern Europe | 4104.86 (-1.48 to 8804.23) | 2.72 (-0.00 to 5.84) | 4928.16 (-2.28 to 10538.91) | 2.60 (-0.00 to 5.57) |
| Eastern Sub-Saharan Africa | 791.22 (-0.11 to 1783.09) | 1.81 (-0.00 to 4.07) | 2503.27 (-0.46 to 5589.09) | 2.47 (-0.00 to 5.51) |
| High-income Asia Pacific | 1052.76 (-0.34 to 2243.97) | 0.93 (-0.00 to 1.99) | 2684.73 (-1.00 to 5830.09) | 1.18 (-0.00 to 2.55) |
| High-income North America | 7382.48 (-3.52 to 15734.52) | 3.92 (-0.00 to 8.35) | 8147.48 (-4.15 to 17459.34) | 2.31 (-0.00 to 4.94) |
| North Africa and Middle East | 914.52 (-0.27 to 1988.20) | 0.89 (-0.00 to 1.92) | 3901.04 (-1.14 to 8462.31) | 1.43 (-0.00 to 3.09) |
| Oceania | 43.16 (-0.01 to 97.65) | 2.36 (-0.00 to 5.32) | 127.00 (-0.03 to 290.68) | 2.62 (-0.00 to 5.97) |
| South Asia | 1897.72 (-0.18 to 4241.89) | 0.54 (-0.00 to 1.21) | 7157.56 (-0.75 to 16140.44) | 0.83 (-0.00 to 1.88) |
| Southeast Asia | 2125.17 (-0.32 to 4724.12) | 1.35 (-0.00 to 3.00) | 7646.64 (-2.11 to 17229.32) | 1.98 (-0.00 to 4.47) |
| Southern Latin America | 979.05 (-0.24 to 2097.44) | 3.91 (-0.00 to 8.40) | 1378.68 (-0.45 to 3024.36) | 2.88 (-0.00 to 6.31) |
| Southern Sub-Saharan Africa | 368.34 (-0.18 to 827.25) | 2.41 (-0.00 to 5.43) | 1153.66 (-0.53 to 2545.84) | 3.63 (-0.00 to 8.01) |
| Tropical Latin America | 1161.83 (-0.59 to -0.59) | 2.22 (-0.00 to 4.76) | 3336.36 (-1.63 to 7178.80) | 2.33 (-0.00 to 5.02) |
| Western Europe | 12566.38 (-4.27 to 26874.21) | 4.12 (-0.00 to 8.79) | 12823.73 (-6.62 to 27509.41) | 2.45 (-0.00 to 5.25) |
| Western Sub-Saharan Africa | 846.62 (-0.17 to 1806.86) | 1.68 (-0.00 to 3.58) | 3105.038 (-0.60 to 6982.94) | 2.62 (-0.00 to 5.88) |
| Countries and territories |  |  |  |  |
| Afghanistan | 46.08(-0.00, 141.30) | 1.17(-0.00, 3.55) | 127.06(-0.00, 378.06) | 1.78(-0.00, 5.04) |
| American Samoa | 14.35(-0.00, 31.84) | 1.24(-0.00, 2.76) | 31.30(-0.00, 70.13) | 1.41(-0.00, 3.15) |
| Angola | 58.77(-0.00, 137.40) | 0.88(-0.00, 2.07) | 204.22(-0.00, 486.93) | 1.00(-0.00, 2.39) |
| Antigua and Barbuda | 0.43(-0.00, 1.02) | 3.44(-0.00, 8.09) | 1.34(-0.00, 3.16) | 5.12(-0.00, 12.08) |
| Arab Republic of Egypt | 0.92(-0.00, 2.16) | 2.99(-0.00, 7.02) | 1.91(-0.00, 4.44) | 2.20(-0.00, 5.10) |
| Argentine Republic | 39.05(-0.00, 96.98) | 1.58(-0.00, 3.93) | 187.65(-0.00, 458.93) | 2.53(-0.00, 6.20) |
| Australia | 1.04(-0.00, 2.26) | 3.56(-0.00, 7.76) | 2.37(-0.00, 5.13) | 4.07(-0.00, 8.83) |
| Bangladesh | 742.39(-0.00, 1604.22) | 4.26(-0.00, 9.22) | 1007.61(-0.02, 2226.94) | 3.28(-0.00, 7.24) |
| Barbados | 61.88(-0.00, 131.63) | 3.94(-0.00, 8.40) | 68.59(-0.00, 145.26) | 2.87(-0.00, 6.09) |
| Belize | 357.41(-0.00, 778.09) | 3.42(-0.00, 7.46) | 493.12(-0.00, 1075.22) | 2.01(-0.00, 4.37) |
| Benin | 256.03(-0.00, 554.22) | 4.06(-0.00, 8.79) | 237.26(-0.00, 525.26) | 2.28(-0.00, 5.04) |
| Bermuda | 67.98(-0.00, 149.14) | 2.38(-0.00, 5.24) | 118.31(-0.00, 270.04) | 1.92(-0.00, 4.38) |
| Bhutan | 4.39(-0.00, 9.52) | 4.82(-0.00, 10.43) | 11.64(-0.00, 25.90) | 5.10(-0.00, 11.31) |
| Bolivarian Republic of Venezuela | 3.00(-0.00, 6.95) | 2.79(-0.00, 6.48) | 13.32(-0.00, 31.07) | 2.58(-0.00, 6.09) |
| Bosnia and Herzegovina | 79.30(-0.00, 197.18) | 0.24(-0.00, 0.60) | 359.09(-0.00, 898.28) | 0.42(-0.00, 1.05) |
| Botswana | 6.70(-0.00, 14.32) | 4.46(-0.00, 9.55) | 13.15(-0.00, 29.54) | 4.80(-0.00, 10.80) |
| Brunei Darussalam | 170.81(-0.00, 374.35) | 2.45(-0.00, 5.35) | 186.87(-0.00, 420.33) | 2.17(-0.00, 4.87) |
| Burkina Faso | 401.81(-0.00, 867.29) | 4.96(-0.00, 10.70) | 354.01(-0.00, 780.27) | 2.69(-0.00, 5.92) |
| Burundi | 0.62(-0.00, 1.37) | 1.19(-0.00, 2.60) | 2.87(-0.00, 6.41) | 1.60(-0.00, 3.57) |
| Cabo Verde | 14.06(-0.00, 33.30) | 1.21(-0.00, 2.88) | 41.33(-0.00, 104.19) | 1.33(-0.00, 3.35) |
| Cameroon | 1.73(-0.00, 3.75) | 5.17(-0.00, 11.18) | 2.08(-0.00, 4.69) | 2.77(-0.00, 6.26) |
| Canada | 0.99(-0.00, 2.58) | 0.63(-0.00, 1.64) | 2.76(-0.00, 7.01) | 0.77(-0.00, 1.97) |
| Central African Republic | 38.70(-0.00, 99.22) | 2.07(-0.00, 5.30) | 122.57(-0.00, 304.18) | 2.38(-0.00, 5.92) |
| Chad | 40.46(-0.00, 91.22) | 1.72(-0.00, 3.88) | 77.16(-0.00, 178.56) | 2.30(-0.00, 5.33) |
| Commonwealth of Dominica | 7.33(-0.00, 18.14) | 2.41(-0.00, 5.91) | 24.93(-0.00, 61.51) | 3.06(-0.00, 7.43) |
| Commonwealth of the Bahamas | 1140.90(-0.58, 2444.74) | 2.23(-0.00, 4.79) | 3258.71(-1.60, 7010.51) | 2.33(-0.00, 5.02) |
| Comoros | 1.47(-0.00, 3.43) | 2.03(-0.00, 4.71) | 5.21(-0.00, 12.21) | 2.28(-0.00, 5.33) |
| Congo | 178.94(-0.00, 387.80) | 2.82(-0.00, 6.11) | 241.05(-0.00, 538.87) | 3.27(-0.00, 7.32) |
| Cook Islands | 59.59(-0.00, 139.36) | 2.47(-0.00, 5.79) | 153.78(-0.00, 366.40) | 2.88(-0.00, 6.84) |
| Czech Republic | 15.04(-0.00, 37.05) | 1.10(-0.00, 2.72) | 22.54(-0.00, 58.56) | 0.75(-0.00, 1.96) |
| Côte d'Ivoire | 2.29(-0.00, 5.38) | 1.94(-0.00, 4.58) | 4.98(-0.00, 11.90) | 1.98(-0.00, 4.71) |
| Democratic People's Republic of Korea | 49.59(-0.00, 131.34) | 1.78(-0.00, 4.69) | 198.23(-0.00, 473.59) | 2.72(-0.00, 6.49) |
| Democratic Republic of the Congo | 49.65(-0.00, 114.47) | 1.89(-0.00, 4.36) | 170.87(-0.00, 428.62) | 2.25(-0.00, 5.60) |
| Democratic Republic of Timor-Leste | 658.86(-0.00, 1421.47) | 3.73(-0.00, 8.06) | 837.17(-0.01, 1868.46) | 2.12(-0.00, 4.74) |
| Democratic Socialist Republic of Sri Lanka | 14.86(-0.00, 36.42) | 2.19(-0.00, 5.35) | 34.48(-0.00, 85.32) | 2.55(-0.00, 6.29) |
| Djibouti | 19.13(-0.00, 45.03) | 1.20(-0.00, 2.83) | 52.12(-0.00, 125.64) | 1.46(-0.00, 3.52) |
| Dominican Republic | 133.18(-0.00, 288.86) | 2.42(-0.00, 5.25) | 243.10(-0.00, 535.90) | 1.73(-0.00, 3.82) |
| Eastern Republic of Uruguay | 5575.79(-2.29, 12294.93) | 1.15(-0.00, 2.54) | 12530.06(-4.96, 28090.30) | 1.09(-0.00, 2.45) |
| Equatorial Guinea | 193.30(-0.00, 418.13) | 1.87(-0.00, 4.05) | 580.29(-0.00, 1295.86) | 1.90(-0.00, 4.24) |
| Eritrea | 2.22(-0.00, 5.23) | 1.90(-0.00, 4.49) | 6.97(-0.00, 17.41) | 2.45(-0.00, 6.14) |
| Eswatini | 18.14(-0.00, 51.65) | 2.94(-0.00, 8.21) | 62.73(-0.00, 168.62) | 3.63(-0.00, 9.57) |
| Ethiopia | 0.32(-0.00, 0.75) | 4.81(-0.00, 11.20) | 0.69(-0.00, 1.63) | 5.10(-0.00, 12.01) |
| Federal Republic of Germany | 16.12(-0.00, 35.29) | 1.60(-0.00, 3.51) | 67.03(-0.00, 148.31) | 2.20(-0.00, 4.87) |
| Federated States of Micronesia | 49.30(-0.00, 113.02) | 2.05(-0.00, 4.68) | 181.41(-0.00, 429.53) | 2.66(-0.00, 6.31) |
| Federative Republic of Brazil | 113.17(-0.00, 244.95) | 3.54(-0.00, 7.66) | 130.46(-0.00, 282.60) | 2.66(-0.00, 5.77) |
| French Republic | 135.58(-0.00, 291.77) | 2.43(-0.00, 5.23) | 250.98(-0.00, 559.02) | 2.33(-0.00, 5.20) |
| Gabon | 13.83(-0.00, 32.34) | 3.42(-0.00, 8.18) | 29.13(-0.00, 66.26) | 2.83(-0.00, 6.47) |
| Gambia | 266.52(-0.00, 576.55) | 3.58(-0.00, 7.75) | 270.73(-0.00, 592.46) | 2.29(-0.00, 5.01) |
| Georgia | 127.44(-0.00, 316.06) | 1.31(-0.00, 3.26) | 259.11(-0.00, 646.55) | 1.39(-0.00, 3.48) |
| Ghana | 103.28(-0.00, 262.24) | 1.06(-0.00, 2.72) | 246.78(-0.00, 660.86) | 1.09(-0.00, 2.91) |
| Grand Duchy of Luxembourg | 237.06(-0.00, 510.73) | 5.55(-0.00, 11.99) | 181.23(-0.00, 398.13) | 2.70(-0.00, 5.92) |
| Greenland | 2.02(-0.00, 4.75) | 2.41(-0.00, 5.67) | 11.12(-0.00, 27.24) | 2.98(-0.00, 7.20) |
| Grenada | 1.29(-0.00, 2.97) | 4.08(-0.00, 9.37) | 1.86(-0.00, 4.27) | 4.19(-0.00, 9.66) |
| Guam | 31.62(-0.00, 71.06) | 1.50(-0.00, 3.38) | 100.78(-0.00, 237.06) | 1.79(-0.00, 4.20) |
| Guinea | 36.08(-0.00, 78.26) | 1.17(-0.00, 2.53) | 150.57(-0.00, 334.93) | 1.67(-0.00, 3.70) |
| Guinea-Bissau | 184.17(-0.00, 411.90) | 1.03(-0.00, 2.30) | 808.37(-0.00, 1863.00) | 2.13(-0.00, 4.90) |
| Hashemite Kingdom of Jordan | 13.90(-0.00, 31.47) | 0.80(-0.00, 1.82) | 50.59(-0.00, 119.08) | 1.49(-0.00, 3.50) |
| Hellenic Republic | 2.20(-0.00, 5.60) | 1.92(-0.00, 4.87) | 11.06(-0.00, 28.72) | 3.48(-0.00, 8.94) |
| Hungary | 16.00(-0.00, 40.57) | 2.21(-0.00, 5.59) | 51.73(-0.00, 131.06) | 3.12(-0.00, 7.88) |
| Independent State of Papua New Guinea | 34.71(-0.00, 75.26) | 3.14(-0.00, 6.82) | 33.99(-0.00, 76.84) | 2.26(-0.00, 5.10) |
| Independent State of Samoa | 3.93(-0.00, 9.25) | 2.48(-0.00, 5.85) | 12.29(-0.00, 32.27) | 3.96(-0.00, 10.31) |
| India | 224.04(-0.02, 579.43) | 1.94(-0.00, 4.91) | 548.67(-0.08, 1255.57) | 2.12(-0.00, 4.86) |
| Ireland | 9.05(-0.00, 21.60) | 4.05(-0.00, 9.63) | 21.36(-0.00, 50.45) | 5.22(-0.00, 12.35) |
| Islamic Republic of Iran | 124.12(-0.00, 267.85) | 3.26(-0.00, 7.04) | 142.15(-0.00, 312.81) | 2.05(-0.00, 4.52) |
| Jamaica | 1775.12(-0.01, 3826.23) | 4.01(-0.00, 8.63) | 2104.37(-0.05, 4602.97) | 2.63(-0.00, 5.75) |
| Japan | 9.08(-0.00, 22.13) | 2.93(-0.00, 7.16) | 21.51(-0.00, 52.94) | 3.64(-0.00, 8.88) |
| Kenya | 1.32(-0.00, 3.12) | 0.64(-0.00, 1.50) | 5.61(-0.00, 14.24) | 0.95(-0.00, 2.40) |
| Kingdom of Bahrain | 130.80(-0.00, 282.59) | 3.85(-0.00, 8.31) | 128.36(-0.00, 283.25) | 4.02(-0.00, 8.85) |
| Kingdom of Belgium | 2831.39(-0.01, 6144.19) | 4.17(-0.00, 9.03) | 2855.81(-0.04, 6243.95) | 2.66(-0.00, 5.82) |
| Kingdom of Cambodia | 67.98(-0.00, 156.66) | 1.73(-0.00, 4.02) | 236.25(-0.00, 578.04) | 2.32(-0.00, 5.69) |
| Kingdom of Denmark | 273.31(-0.00, 592.66) | 3.43(-0.00, 7.44) | 397.92(-0.00, 861.36) | 2.91(-0.00, 6.28) |
| Kingdom of Norway | 0.79(-0.00, 1.79) | 4.10(-0.00, 9.25) | 0.78(-0.00, 1.85) | 2.07(-0.00, 4.88) |
| Kingdom of Spain | 1.12(-0.00, 2.54) | 2.99(-0.00, 6.73) | 2.34(-0.00, 5.30) | 3.89(-0.00, 8.83) |
| Kingdom of Sweden | 0.90(-0.00, 2.04) | 2.38(-0.00, 5.43) | 1.93(-0.00, 4.49) | 1.73(-0.00, 4.01) |
| Kingdom of Thailand | 12.03(-0.00, 26.73) | 0.59(-0.00, 1.33) | 64.63(-0.00, 147.10) | 1.02(-0.00, 2.32) |
| Kingdom of the Netherlands | 19.46(-0.00, 47.73) | 1.02(-0.00, 2.50) | 61.48(-0.00, 149.73) | 1.77(-0.00, 4.33) |
| Kingdom of Tonga | 4.69(-0.00, 11.59) | 1.93(-0.00, 4.75) | 12.56(-0.00, 31.25) | 2.67(-0.00, 6.64) |
| Kyrgyz Republic | 4.16(-0.00, 9.46) | 1.82(-0.00, 4.16) | 8.71(-0.00, 21.27) | 2.32(-0.00, 5.67) |
| Lao People's Democratic Republic | 46.79(-0.00, 130.61) | 2.44(-0.00, 6.69) | 130.13(-0.00, 341.71) | 2.88(-0.00, 7.53) |
| Lebanon | 11.55(-0.00, 28.35) | 0.94(-0.00, 2.34) | 61.07(-0.00, 149.39) | 1.66(-0.00, 4.05) |
| Lesotho | 294.60(-0.00, 636.21) | 3.82(-0.00, 8.26) | 304.33(-0.00, 660.90) | 2.88(-0.00, 6.26) |
| Liberia | 5.50(-0.00, 11.92) | 3.57(-0.00, 7.74) | 7.11(-0.00, 15.60) | 2.22(-0.00, 4.86) |
| Libya | 1047.37(-0.04, 2408.76) | 0.37(-0.00, 0.85) | 4053.12(-0.16, 9411.87) | 0.60(-0.00, 1.39) |
| Madagascar | 683.70(-0.05, 1748.58) | 1.08(-0.00, 2.78) | 2423.52(-0.18, 6352.19) | 1.63(-0.00, 4.30) |
| Malawi | 121.98(-0.03, 269.72) | 0.77(-0.00, 1.69) | 515.89(-0.13, 1119.04) | 1.08(-0.00, 2.36) |
| Malaysia | 63.63(-0.00, 148.88) | 1.29(-0.00, 3.02) | 229.03(-0.00, 579.77) | 1.47(-0.00, 3.75) |
| Mali | 96.36(-0.00, 208.37) | 4.54(-0.00, 9.83) | 98.78(-0.00, 216.75) | 2.30(-0.00, 5.04) |
| Mauritania | 104.90(-0.00, 227.54) | 4.13(-0.00, 8.96) | 173.88(-0.00, 380.42) | 2.52(-0.00, 5.51) |
| Mauritius | 1783.38(-1.00, 3813.17) | 3.85(-0.00, 8.24) | 2000.45(-1.03, 4303.08) | 2.45(-0.00, 5.24) |
| Monaco | 24.92(-0.00, 54.10) | 2.60(-0.00, 5.64) | 59.74(-0.00, 136.12) | 3.44(-0.00, 7.85) |
| Mongolia | 886.30(-0.27, 1888.22) | 0.96(-0.00, 2.05) | 2223.94(-0.76, 4845.12) | 1.31(-0.00, 2.83) |
| Montenegro | 14.96(-0.00, 35.39) | 1.72(-0.00, 4.07) | 82.78(-0.00, 195.13) | 1.80(-0.00, 4.24) |
| Morocco | 223.89(-0.00, 491.23) | 3.12(-0.00, 6.85) | 188.46(-0.00, 423.41) | 1.87(-0.00, 4.20) |
| Mozambique | 73.60(-0.01, 175.70) | 1.51(-0.00, 3.60) | 353.27(-0.09, 827.88) | 2.61(-0.00, 6.05) |
| Namibia | 0.66(-0.00, 1.58) | 3.08(-0.00, 7.39) | 1.77(-0.00, 4.24) | 4.31(-0.00, 10.28) |
| Nauru | 4.67(-0.00, 10.18) | 1.19(-0.00, 2.59) | 22.60(-0.00, 49.50) | 1.09(-0.00, 2.39) |
| Nepal | 41.70(-0.00, 91.38) | 2.52(-0.00, 5.51) | 45.86(-0.00, 101.14) | 1.59(-0.00, 3.51) |
| New Zealand | 20.80(-0.00, 54.44) | 1.67(-0.00, 4.35) | 65.98(-0.00, 157.83) | 2.30(-0.00, 5.51) |
| Niger | 57.19(-0.00, 125.10) | 2.96(-0.00, 6.49) | 57.79(-0.00, 128.61) | 2.69(-0.00, 5.98) |
| Nigeria | 30.26(-0.00, 73.39) | 2.54(-0.00, 6.11) | 91.22(-0.00, 208.20) | 2.72(-0.00, 6.21) |
| Niue | 9.12(-0.00, 22.44) | 2.00(-0.00, 4.91) | 22.96(-0.00, 58.25) | 3.94(-0.00, 9.97) |
| North Macedonia | 6.94(-0.00, 16.45) | 1.06(-0.00, 2.51) | 18.89(-0.00, 48.42) | 1.34(-0.00, 3.44) |
| Northern Mariana Islands | 11.35(-0.00, 26.47) | 1.01(-0.00, 2.36) | 52.77(-0.00, 126.95) | 1.56(-0.00, 3.75) |
| Oman | 64.54(-0.00, 140.10) | 2.65(-0.00, 5.75) | 77.70(-0.00, 176.03) | 2.53(-0.00, 5.72) |
| Pakistan | 13.63(-0.00, 29.63) | 4.69(-0.00, 10.19) | 14.28(-0.00, 31.35) | 2.36(-0.00, 5.18) |
| Palau | 64.22(-0.00, 152.48) | 2.14(-0.00, 5.07) | 163.56(-0.00, 395.40) | 2.28(-0.00, 5.50) |
| Palestine | 21.35(-0.00, 51.05) | 0.92(-0.00, 2.21) | 93.55(-0.00, 233.02) | 2.08(-0.00, 5.18) |
| People's Democratic Republic of Algeria | 143.33(-0.00, 332.65) | 2.47(-0.00, 5.74) | 501.81(-0.00, 1158.93) | 3.06(-0.00, 7.08) |
| People's Republic of China | 0.16(-0.00, 0.50) | 0.27(-0.00, 0.83) | 1.21(-0.00, 3.01) | 0.55(-0.00, 1.39) |
| Plurinational State of Bolivia | 43.29(-0.00, 99.39) | 1.82(-0.00, 4.18) | 112.41(-0.00, 270.23) | 2.08(-0.00, 4.96) |
| Portuguese Republic | 11.08(-0.00, 24.20) | 4.78(-0.00, 10.46) | 13.62(-0.00, 30.40) | 2.66(-0.00, 5.91) |
| Principality of Andorra | 0.26(-0.00, 0.64) | 2.74(-0.00, 6.80) | 0.80(-0.00, 2.13) | 3.73(-0.00, 9.76) |
| Puerto Rico | 10.77(-0.00, 25.90) | 1.95(-0.00, 4.66) | 28.35(-0.00, 67.47) | 2.28(-0.00, 5.43) |
| Qatar | 6.43(-0.00, 13.86) | 1.54(-0.00, 3.34) | 29.61(-0.00, 64.23) | 2.97(-0.00, 6.45) |
| Republic of Albania | 383.86(-0.16, 822.41) | 1.53(-0.00, 3.28) | 1290.85(-0.59, 2802.33) | 1.79(-0.00, 3.89) |
| Republic of Armenia | 0.90(-0.00, 2.20) | 3.31(-0.00, 8.07) | 1.80(-0.00, 4.39) | 4.31(-0.00, 10.44) |
| Republic of Austria | 1.67(-0.00, 3.95) | 4.62(-0.00, 10.86) | 2.40(-0.00, 5.57) | 4.69(-0.00, 10.90) |
| Republic of Azerbaijan | 4.43(-0.00, 10.38) | 0.72(-0.00, 1.68) | 11.52(-0.00, 26.00) | 0.81(-0.00, 1.82) |
| Republic of Belarus | 10.80(-0.00, 24.41) | 3.12(-0.00, 7.05) | 18.57(-0.00, 41.18) | 3.66(-0.00, 8.13) |
| Republic of Bulgaria | 59.11(-0.00, 139.43) | 0.70(-0.00, 1.64) | 237.78(-0.00, 594.71) | 1.18(-0.00, 2.94) |
| Republic of Chile | 38.97(-0.00, 93.89) | 1.16(-0.00, 2.80) | 143.78(-0.00, 358.83) | 2.25(-0.00, 5.64) |
| Republic of Colombia | 198.39(-0.00, 504.84) | 1.34(-0.00, 3.41) | 701.19(-0.00, 1671.92) | 2.42(-0.00, 5.78) |
| Republic of Costa Rica | 9.21(-0.00, 21.81) | 2.54(-0.00, 6.00) | 36.40(-0.00, 89.22) | 4.52(-0.00, 10.98) |
| Republic of Croatia | 0.10(-0.00, 0.27) | 3.43(-0.00, 9.13) | 0.18(-0.00, 0.48) | 5.15(-0.00, 13.87) |
| Republic of Cuba | 52.56(-0.00, 124.30) | 0.89(-0.00, 2.13) | 153.93(-0.00, 371.82) | 1.15(-0.00, 2.78) |
| Republic of Cyprus | 510.31(-0.00, 1118.11) | 4.74(-0.00, 10.38) | 516.72(-0.00, 1130.78) | 2.71(-0.00, 5.92) |
| Republic of Ecuador | 92.01(-0.00, 199.18) | 4.42(-0.00, 9.57) | 104.25(-0.01, 226.38) | 2.35(-0.00, 5.09) |
| Republic of El Salvador | 6.97(-0.00, 15.94) | 0.73(-0.00, 1.67) | 28.11(-0.00, 66.94) | 0.98(-0.00, 2.33) |
| Republic of Estonia | 17.75(-0.00, 41.96) | 1.07(-0.00, 2.52) | 59.42(-0.00, 146.41) | 1.24(-0.00, 3.03) |
| Republic of Fiji | 437.49(-0.04, 985.48) | 1.76(-0.00, 3.97) | 1804.80(-0.22, 4258.41) | 3.26(-0.00, 7.68) |
| Republic of Finland | 0.05(-0.00, 0.11) | 3.83(-0.00, 9.05) | 0.05(-0.00, 0.13) | 4.60(-0.00, 11.01) |
| Republic of Guatemala | 32.02(-0.00, 69.66) | 3.00(-0.00, 6.54) | 56.89(-0.00, 128.64) | 3.43(-0.00, 7.78) |
| Republic of Guyana | 0.34(-0.00, 0.80) | 3.02(-0.00, 7.17) | 0.99(-0.00, 2.30) | 3.59(-0.00, 8.33) |
| Republic of Haiti | 116.88(-0.05, 251.10) | 3.28(-0.00, 7.02) | 100.32(-0.05, 215.34) | 1.78(-0.00, 3.82) |
| Republic of Honduras | 1.93(-0.00, 4.61) | 0.48(-0.00, 1.13) | 6.49(-0.00, 15.30) | 0.53(-0.00, 1.27) |
| Republic of Iceland | 717.49(-0.15, 1656.47) | 2.15(-0.00, 5.00) | 2588.67(-0.59, 6033.30) | 3.39(-0.00, 7.88) |
| Republic of Indonesia | 0.29(-0.00, 0.70) | 5.47(-0.00, 13.36) | 0.59(-0.00, 1.37) | 5.32(-0.00, 12.38) |
| Republic of Iraq | 10.99(-0.00, 27.19) | 2.20(-0.00, 5.44) | 35.39(-0.00, 82.00) | 2.34(-0.00, 5.43) |
| Republic of Italy | 12.42(-0.00, 27.31) | 1.46(-0.00, 3.21) | 45.47(-0.00, 101.47) | 1.85(-0.00, 4.13) |
| Republic of Kazakhstan | 22.76(-0.00, 55.45) | 1.83(-0.00, 4.44) | 77.07(-0.00, 188.24) | 2.07(-0.00, 5.05) |
| Republic of Kiribati | 20.93(-0.00, 48.00) | 1.64(-0.00, 3.76) | 77.65(-0.00, 186.56) | 2.36(-0.00, 5.67) |
| Republic of Korea | 104.78(-0.00, 236.77) | 1.47(-0.00, 3.33) | 276.10(-0.00, 664.85) | 1.45(-0.00, 3.48) |
| Republic of Latvia | 415.61(-0.11, 914.41) | 2.35(-0.00, 5.18) | 1531.51(-0.47, 3391.52) | 3.12(-0.00, 6.92) |
| Republic of Lithuania | 691.62(-0.38, 1470.96) | 2.96(-0.00, 6.30) | 1174.86(-0.65, 2483.15) | 2.98(-0.00, 6.30) |
| Republic of Maldives | 249.64(-0.00, 545.14) | 3.56(-0.00, 7.77) | 287.84(-0.00, 630.92) | 2.17(-0.00, 4.74) |
| Republic of Malta | 48.72(-0.00, 105.27) | 2.52(-0.00, 5.44) | 79.44(-0.00, 177.22) | 2.15(-0.00, 4.80) |
| Republic of Moldova | 1.67(-0.00, 3.89) | 2.16(-0.00, 5.07) | 11.25(-0.00, 26.44) | 2.08(-0.00, 4.88) |
| Republic of Nicaragua | 138.19(-0.00, 303.13) | 0.73(-0.00, 1.60) | 389.39(-0.00, 842.36) | 0.81(-0.00, 1.74) |
| Republic of Panama | 74.39(-0.00, 161.10) | 3.04(-0.00, 6.58) | 79.83(-0.00, 179.96) | 2.46(-0.00, 5.55) |
| Republic of Paraguay | 363.37(-0.00, 798.43) | 2.42(-0.00, 5.32) | 566.21(-0.01, 1247.33) | 2.85(-0.00, 6.27) |
| Republic of Peru | 2370.59(-1.19, 5035.24) | 2.43(-0.00, 5.17) | 3445.02(-1.59, 7304.99) | 2.67(-0.00, 5.66) |
| Republic of Poland | 33.75(-0.00, 86.36) | 1.97(-0.00, 5.01) | 95.04(-0.00, 233.59) | 2.56(-0.00, 6.34) |
| Republic of Serbia | 1.10(-0.00, 2.40) | 5.74(-0.00, 12.50) | 1.46(-0.00, 3.21) | 4.01(-0.00, 8.83) |
| Republic of Singapore | 1.92(-0.00, 4.19) | 4.23(-0.00, 9.21) | 4.07(-0.00, 9.08) | 3.11(-0.00, 6.94) |
| Republic of Slovenia | 1.45(-0.00, 3.16) | 3.86(-0.00, 8.43) | 2.80(-0.00, 6.22) | 3.69(-0.00, 8.20) |
| Republic of Suriname | 1.05(-0.00, 2.50) | 2.25(-0.00, 5.36) | 2.18(-0.00, 5.30) | 2.67(-0.00, 6.48) |
| Republic of Tajikistan | 0.52(-0.00, 1.17) | 2.75(-0.00, 6.16) | 0.60(-0.00, 1.46) | 1.46(-0.00, 3.59) |
| Republic of the Marshall Islands | 0.21(-0.00, 0.50) | 0.59(-0.00, 1.43) | 0.71(-0.00, 1.87) | 1.02(-0.00, 2.67) |
| Republic of the Philippines | 27.65(-0.00, 64.88) | 0.68(-0.00, 1.60) | 162.32(-0.00, 380.24) | 0.96(-0.00, 2.25) |
| Republic of the Union of Myanmar | 25.93(-0.00, 60.46) | 1.37(-0.00, 3.22) | 92.11(-0.00, 223.73) | 2.03(-0.00, 4.94) |
| Republic of Trinidad and Tobago | 225.32(-0.00, 503.36) | 3.90(-0.00, 8.75) | 318.01(-0.00, 704.16) | 3.60(-0.00, 7.97) |
| Republic of Uzbekistan | 0.52(-0.00, 1.27) | 1.69(-0.00, 4.13) | 1.58(-0.00, 3.75) | 2.39(-0.00, 5.70) |
| Republic of Vanuatu | 6.94(-0.00, 17.04) | 0.59(-0.00, 1.45) | 26.65(-0.00, 67.04) | 1.13(-0.00, 2.85) |
| Romania | 26.80(-0.00, 57.84) | 1.97(-0.00, 4.25) | 66.19(-0.00, 145.66) | 1.39(-0.00, 3.05) |
| Russian Federation | 95.81(-0.00, 211.69) | 2.96(-0.00, 6.56) | 144.20(-0.00, 320.56) | 2.80(-0.00, 6.24) |
| Rwanda | 43.76(-0.00, 95.55) | 3.24(-0.00, 7.08) | 59.20(-0.00, 131.91) | 2.27(-0.00, 5.06) |
| Saint Kitts and Nevis | 1.22(-0.00, 3.03) | 1.47(-0.00, 3.66) | 5.39(-0.00, 13.60) | 2.38(-0.00, 6.01) |
| Saint Lucia | 29.43(-0.00, 72.97) | 1.91(-0.00, 4.70) | 77.08(-0.00, 189.86) | 2.08(-0.00, 5.09) |
| Saint Vincent and the Grenadines | 293.12(-0.17, 676.22) | 2.48(-0.00, 5.74) | 891.71(-0.46, 1980.39) | 3.53(-0.00, 7.83) |
| San Marino | 22.80(-0.00, 55.98) | 1.59(-0.00, 3.90) | 46.61(-0.00, 113.66) | 1.96(-0.00, 4.75) |
| Sao Tome and Principe | 922.66(-0.00, 2029.69) | 3.27(-0.00, 7.19) | 986.68(-0.02, 2169.15) | 1.80(-0.00, 3.95) |
| Saudi Arabia | 27.59(-0.00, 65.76) | 0.43(-0.00, 1.02) | 83.56(-0.00, 220.51) | 0.57(-0.00, 1.49) |
| Senegal | 35.03(-0.00, 94.31) | 0.62(-0.00, 1.64) | 121.21(-0.00, 322.20) | 0.90(-0.00, 2.35) |
| Seychelles | 2.78(-0.00, 6.36) | 1.89(-0.00, 4.33) | 7.26(-0.00, 17.44) | 2.06(-0.00, 4.95) |
| Sierra Leone | 227.86(-0.04, 494.37) | 2.96(-0.00, 6.41) | 220.32(-0.06, 486.80) | 1.81(-0.00, 4.00) |
| Slovak Republic | 195.07(-0.00, 424.15) | 3.48(-0.00, 7.58) | 204.79(-0.00, 451.84) | 1.96(-0.00, 4.30) |
| Socialist Republic of Viet Nam | 34.41(-0.00, 79.30) | 1.05(-0.00, 2.42) | 102.55(-0.00, 244.12) | 1.31(-0.00, 3.11) |
| Solomon Islands | 106.42(-0.00, 231.15) | 1.14(-0.00, 2.47) | 389.43(-0.00, 851.10) | 1.74(-0.00, 3.81) |
| Somalia | 29.04(-0.00, 65.63) | 1.83(-0.00, 4.15) | 49.43(-0.00, 123.89) | 1.29(-0.00, 3.20) |
| South Africa | 310.24(-0.00, 712.19) | 1.38(-0.00, 3.18) | 1216.52(-0.00, 2823.35) | 2.09(-0.00, 4.85) |
| South Sudan | 2.01(-0.00, 5.17) | 1.06(-0.00, 2.71) | 7.66(-0.00, 18.84) | 1.57(-0.00, 3.88) |
| State of Israel | 9.80(-0.00, 23.52) | 1.24(-0.00, 2.99) | 41.28(-0.00, 105.39) | 1.75(-0.00, 4.48) |
| State of Kuwait | 0.02(-0.00, 0.06) | 3.56(-0.00, 8.89) | 0.03(-0.00, 0.08) | 4.05(-0.00, 9.92) |
| Sudan | 1.32(-0.00, 3.02) | 4.27(-0.00, 9.81) | 2.21(-0.00, 5.33) | 4.92(-0.00, 11.91) |
| Swiss Confederation | 15.28(-0.00, 33.43) | 3.38(-0.00, 7.43) | 33.90(-0.00, 78.50) | 3.25(-0.00, 7.54) |
| Syrian Arab Republic | 32.63(-0.00, 74.94) | 1.16(-0.00, 2.68) | 103.24(-0.00, 245.75) | 1.39(-0.00, 3.31) |
| Taiwan (Province of China) | 150.05(-0.00, 345.29) | 0.72(-0.00, 1.65) | 843.44(-0.00, 1928.19) | 1.63(-0.00, 3.72) |
| Togo | 21.32(-0.00, 45.80) | 1.90(-0.00, 4.09) | 41.54(-0.00, 95.34) | 1.67(-0.00, 3.83) |
| Tokelau | 0.11(-0.00, 0.29) | 3.18(-0.00, 8.01) | 0.21(-0.00, 0.50) | 3.68(-0.00, 8.93) |
| Tunisia | 80.26(-0.00, 186.47) | 2.19(-0.00, 5.08) | 317.15(-0.00, 755.61) | 3.53(-0.00, 8.38) |
| Turkey | 1332.65(-0.05, 2914.55) | 3.49(-0.00, 7.63) | 1046.97(-0.08, 2477.18) | 2.53(-0.00, 6.02) |
| Turkmenistan | 5.97(-0.00, 13.95) | 2.05(-0.00, 4.79) | 45.61(-0.00, 108.38) | 2.18(-0.00, 5.16) |
| Tuvalu | 2403.01(-0.98, 5115.72) | 5.10(-0.00, 10.86) | 1880.85(-0.71, 4057.50) | 2.60(-0.00, 5.60) |
| Uganda | 130.74(-0.00, 309.10) | 2.15(-0.00, 5.10) | 395.39(-0.00, 964.07) | 2.62(-0.00, 6.39) |
| Ukraine | 6722.65(-3.21, 14353.55) | 3.94(-0.00, 8.42) | 7309.40(-3.75, 15646.59) | 2.33(-0.00, 4.99) |
| United Arab Emirates | 1.90(-0.00, 4.30) | 4.06(-0.00, 9.24) | 2.47(-0.00, 6.09) | 2.85(-0.00, 7.08) |
| United Kingdom of Great Britain and Northern Ireland | 103.44(-0.00, 224.88) | 4.99(-0.00, 10.85) | 127.89(-0.00, 280.87) | 4.18(-0.00, 9.17) |
| United Mexican States | 122.08(-0.00, 266.13) | 1.86(-0.00, 4.06) | 252.94(-0.00, 563.10) | 1.53(-0.00, 3.39) |
| United Republic of Tanzania | 0.62(-0.00, 1.51) | 1.70(-0.00, 4.12) | 2.72(-0.00, 6.46) | 2.66(-0.00, 6.31) |
| United States of America | 107.18(-0.00, 233.85) | 1.84(-0.00, 4.03) | 444.11(-0.00, 1008.99) | 2.65(-0.00, 6.01) |
| United States Virgin Islands | 263.70(-0.00, 607.79) | 1.17(-0.00, 2.70) | 873.60(-0.03, 2106.43) | 1.54(-0.00, 3.69) |
| Yemen | 15.70(-0.00, 39.67) | 0.52(-0.00, 1.31) | 80.87(-0.00, 202.63) | 0.85(-0.00, 2.12) |
| Zambia | 36.22(-0.00, 88.83) | 2.10(-0.00, 5.09) | 174.64(-0.00, 464.84) | 3.83(-0.00, 9.95) |
| Zimbabwe | 45.63(-0.00, 107.46) | 2.03(-0.00, 4.78) | 165.37(-0.00, 398.27) | 3.91(-0.00, 9.34) |

**Abbreviation:** ASR, age-standardized rate; EAPC, estimated annual percentage change; CI, confidence interval; UI, uncertainty interval; SDI, socio-demographic index.

**Table S2. YLLs and YLDs Trends for Breast Cancer Associated with High Red Meat Diets, 1990–2021**

| **Characteristics** | **YLLs** | | | **YLDs** | | |
| --- | --- | --- | --- | --- | --- | --- |
|  | **2021** | | **1990–2021** | **2021** | | **1990–2021** |
|  | **Number (95% UI)** | **ASR (95% UI)** | **EAPC (95% CI)** | **Number (95% UI)** | **ASR (95% UI)** | **EAPC (95% CI)** |
| Global | 2263019.99 (-746.50 to 4893977.19) | 47.42 (-0.02 to 102.56) | -0.72 (-0.77 to -0.66) | 188698.65 (-81.20 to 447393.84) | 3.95 (-0.00 to 9.37) | 0.24 (0.19 to 0.28) |
| Sociodemographic index |  |  |  |  |  |  |
| Low SDI | 167947.84 (-36.66 to 365313.67) | 46.83 (-0.01 to 101.98) | 0.76 (0.64 to 0.89) | 4804.37 (-1.20 to 11394.83) | 1.37 (-0.00 to 3.25) | 1.38 (1.23 to 1.54) |
| Low-middle SDI | 382183.33 (-112.66 to 832430.28) | 41.81 (-0.01 to 91.08) | 1.53 (1.49 to 1.56) | 14722.64 (-4.69 to 34850.96) | 1.63 (-0.00 to 3.87) | 2.52 (2.48 to 2.55) |
| Middle SDI | 666615.45 (-215.44 to 1447540.73) | 42.40 (-0.01 to 92.15) | 0.17 (0.12 to 0.23) | 43703.89 (-17.44 to 102517.12) | 2.79 (-0.00 to 6.53) | 2.07 (2.02 to 2.13) |
| High-middle SDI | 523993.56 (-213.33 to 1128203.86) | 50.09 (-0.02 to 107.93) | -1.14 (-1.24 to -1.05) | 49505.29 (-21.74 to 117071.11) | 4.71 (-0.00 to 11.13) | 0.79 (0.74 to 0.85) |
| High SDI | 519470.73 (-236.54 to 1110993.13) | 52.41 (-0.02 to 112.05) | -1.76 (-1.80 to -1.71) | 75768.82 (-38.23 to 179867.42) | 7.48 (-0.00 to 17.81) | -0.20 (-0.32 to-0.09) |
| GBD regions |  |  |  |  |  |  |
| Afghanistan | 4877.80(-0.06, 14848.43) | 57.76(-0.00, 169.83) | 1.35(1.27, 1.43) | 174.18(-0.00, 564.77) | 2.22(-0.00, 6.88) | 2.11(1.88, 2.34) |
| American Samoa | 787.84(-0.03, 1757.52) | 37.37(-0.00, 83.24) | 0.70(0.49, 0.92) | 59.41(-0.00, 144.72) | 2.79(-0.00, 6.81) | 2.54(2.26, 2.82) |
| Angola | 6620.42(-0.15, 15862.80) | 28.65(-0.00, 68.48) | 0.44(0.33, 0.55) | 552.80(-0.01, 1435.10) | 2.43(-0.00, 6.27) | 2.11(1.98, 2.25) |
| Antigua and Barbuda | 41.23(-0.00, 97.52) | 144.60(-0.00, 342.65) | 1.74(1.62, 1.85) | 1.48(-0.00, 3.75) | 5.23(-0.00, 13.30) | 1.65(1.54, 1.75) |
| Arab Republic of Egypt | 46.53(-0.00, 108.40) | 56.92(-0.00, 132.64) | -0.74(-0.92, -0.56) | 6.54(-0.00, 16.40) | 8.08(-0.00, 20.34) | 0.84(0.60, 1.08) |
| Argentine Republic | 6670.22(-0.13, 16360.19) | 74.08(-0.00, 181.33) | 1.56(1.45, 1.67) | 186.49(-0.00, 492.99) | 2.16(-0.00, 5.62) | 2.30(2.14, 2.47) |
| Australia | 65.37(-0.00, 141.50) | 106.01(-0.00, 229.61) | 0.49(0.28, 0.70) | 4.14(-0.00, 9.76) | 6.72(-0.00, 15.86) | 1.40(1.22, 1.58) |
| Bangladesh | 24908.52(-0.48, 54864.42) | 84.15(-0.00, 185.30) | -0.94(-1.08, -0.80) | 1518.64(-0.03, 3569.72) | 5.09(-0.00, 11.98) | 0.34(0.15, 0.52) |
| Barbados | 1709.95(-0.01, 3608.56) | 73.14(-0.00, 154.45) | -1.69(-2.04, -1.34) | 109.04(-0.00, 255.53) | 4.64(-0.00, 10.89) | -0.27(-0.58, 0.04) |
| Belize | 10872.61(-0.15, 23579.36) | 50.34(-0.00, 109.02) | -2.07(-2.14, -2.00) | 1735.85(-0.03, 4127.34) | 7.93(-0.00, 18.90) | 0.08(-0.11, 0.27) |
| Benin | 4678.35(-0.09, 10289.78) | 52.77(-0.00, 115.84) | -2.08(-2.17, -2.00) | 561.69(-0.01, 1340.15) | 6.48(-0.00, 15.53) | -0.56(-0.69, -0.44) |
| Bermuda | 3847.09(-0.01, 8764.48) | 57.63(-0.00, 131.35) | -0.74(-0.87, -0.61) | 188.96(-0.00, 459.03) | 2.88(-0.00, 6.99) | 0.45(0.22, 0.68) |
| Bhutan | 358.40(-0.00, 802.60) | 146.49(-0.00, 327.60) | 0.24(0.11, 0.37) | 18.88(-0.00, 46.18) | 7.79(-0.00, 19.00) | 1.14(0.97, 1.31) |
| Bolivarian Republic of Venezuela | 443.61(-0.00, 1030.36) | 62.41(-0.00, 146.18) | -0.93(-1.14, -0.72) | 48.65(-0.00, 121.85) | 7.40(-0.00, 18.36) | 1.76(1.53, 1.99) |
| Bosnia and Herzegovina | 14149.09(-0.06, 35555.52) | 16.13(-0.00, 40.49) | 1.59(1.42, 1.75) | 542.68(-0.00, 1463.44) | 0.62(-0.00, 1.67) | 3.24(3.07, 3.40) |
| Botswana | 321.44(-0.00, 726.79) | 125.06(-0.00, 283.24) | 0.47(0.28, 0.66) | 21.32(-0.00, 52.35) | 8.15(-0.00, 20.13) | 1.38(1.16, 1.59) |
| Brunei Darussalam | 4866.35(-0.02, 10942.84) | 59.34(-0.00, 133.34) | -1.67(-1.97, -1.37) | 422.03(-0.00, 1023.65) | 5.10(-0.00, 12.40) | 0.06(-0.16, 0.28) |
| Burkina Faso | 7113.29(-0.16, 15672.46) | 64.02(-0.00, 141.10) | -2.31(-2.46, -2.15) | 914.83(-0.02, 2218.11) | 8.27(-0.00, 20.12) | -0.65(-0.87, -0.43) |
| Burundi | 94.22(-0.00, 209.57) | 47.78(-0.00, 106.37) | 1.06(0.76, 1.35) | 4.45(-0.00, 11.06) | 2.30(-0.00, 5.71) | 1.65(1.47, 1.83) |
| Cabo Verde | 1390.03(-0.00, 3519.80) | 37.83(-0.00, 95.44) | -0.01(-0.19, 0.18) | 40.18(-0.00, 109.46) | 1.13(-0.00, 3.06) | 0.61(0.44, 0.79) |
| Cameroon | 44.01(-0.00, 99.53) | 67.68(-0.00, 153.04) | -2.50(-2.74, -2.26) | 5.07(-0.00, 12.40) | 7.75(-0.00, 19.02) | -0.43(-0.59, -0.27) |
| Canada | 91.14(-0.00, 231.07) | 23.76(-0.00, 60.15) | 0.41(0.25, 0.56) | 3.40(-0.00, 9.32) | 0.90(-0.00, 2.44) | 1.84(1.65, 2.03) |
| Central African Republic | 3767.23(-0.05, 9278.02) | 67.34(-0.00, 166.30) | 0.15(0.09, 0.21) | 138.36(-0.00, 366.67) | 2.50(-0.00, 6.60) | 1.45(1.41, 1.50) |
| Chad | 1843.49(-0.01, 4273.61) | 58.68(-0.00, 136.20) | 0.92(0.72, 1.11) | 134.97(-0.00, 337.68) | 4.29(-0.00, 10.74) | 2.59(2.26, 2.93) |
| Commonwealth of Dominica | 792.36(-0.02, 1989.03) | 79.31(-0.00, 196.30) | 1.04(0.68, 1.40) | 25.50(-0.00, 67.27) | 2.62(-0.00, 6.80) | 1.62(1.27, 1.97) |
| Commonwealth of the Bahamas | 96190.29(-47.30, 206817.02) | 67.61(-0.03, 145.39) | -0.08(-0.15, -0.01) | 4897.68(-2.59, 11546.12) | 3.45(-0.00, 8.13) | 1.17(1.07, 1.26) |
| Comoros | 183.53(-0.00, 431.43) | 70.44(-0.00, 165.17) | 0.82(0.57, 1.07) | 10.94(-0.00, 27.69) | 4.37(-0.00, 10.99) | 1.83(1.64, 2.02) |
| Congo | 5725.14(-0.13, 12828.20) | 86.34(-0.00, 193.79) | 0.24(0.12, 0.36) | 473.96(-0.01, 1143.49) | 6.97(-0.00, 16.89) | 1.27(1.16, 1.39) |
| Cook Islands | 5134.87(-0.03, 12333.77) | 79.72(-0.00, 190.36) | 0.32(0.20, 0.44) | 142.30(-0.00, 358.45) | 2.28(-0.00, 5.68) | 0.85(0.78, 0.93) |
| Czech Republic | 803.42(-0.01, 2098.59) | 21.63(-0.00, 56.18) | -1.75(-2.18, -1.32) | 20.60(-0.00, 57.42) | 0.58(-0.00, 1.60) | -1.23(-1.64, -0.82) |
| C么te d'Ivoire | 137.60(-0.00, 328.83) | 50.25(-0.00, 119.85) | -0.37(-0.65, -0.08) | 6.09(-0.00, 15.44) | 2.25(-0.00, 5.67) | 0.92(0.64, 1.21) |
| Democratic People's Republic of Korea | 6546.16(-0.05, 15667.55) | 81.47(-0.00, 194.69) | 1.12(1.03, 1.20) | 220.21(-0.00, 575.14) | 2.77(-0.00, 7.19) | 2.28(2.24, 2.33) |
| Democratic Republic of the Congo | 5875.22(-0.08, 14817.18) | 63.61(-0.00, 159.57) | 0.37(0.29, 0.45) | 173.28(-0.00, 463.84) | 1.94(-0.00, 5.14) | 0.92(0.77, 1.06) |
| Democratic Republic of Timor-Leste | 17800.63(-0.27, 39613.66) | 51.99(-0.00, 115.61) | -2.12(-2.20, -2.05) | 2691.76(-0.04, 6562.41) | 7.69(-0.00, 18.81) | -0.85(-0.99, -0.72) |
| Democratic Socialist Republic of Sri Lanka | 1230.28(-0.06, 3056.82) | 73.10(-0.00, 180.90) | 0.32(0.25, 0.38) | 27.20(-0.00, 73.72) | 1.73(-0.00, 4.63) | 0.51(0.44, 0.57) |
| Djibouti | 1809.96(-0.01, 4377.72) | 43.17(-0.00, 104.13) | 0.63(0.59, 0.66) | 45.91(-0.00, 121.49) | 1.14(-0.00, 2.99) | 0.89(0.82, 0.97) |
| Dominican Republic | 6016.06(-0.10, 13244.61) | 44.52(-0.00, 97.93) | -1.03(-1.13, -0.93) | 491.71(-0.00, 1190.97) | 3.63(-0.00, 8.81) | 1.10(0.98, 1.21) |
| Eastern Republic of Uruguay | 375787.03(-154.00, 847432.94) | 32.76(-0.01, 73.98) | -0.53(-0.64, -0.41) | 38754.11(-16.70, 92561.56) | 3.36(-0.00, 8.04) | 2.68(2.59, 2.78) |
| Equatorial Guinea | 16834.28(-0.13, 37596.16) | 55.43(-0.00, 123.81) | 0.03(-0.21, 0.27) | 1584.37(-0.00, 3851.36) | 5.23(-0.00, 12.70) | 2.13(1.95, 2.32) |
| Eritrea | 228.94(-0.00, 570.64) | 70.71(-0.00, 176.51) | 0.39(0.22, 0.56) | 6.62(-0.00, 17.90) | 2.09(-0.00, 5.63) | 1.06(0.94, 1.18) |
| Eswatini | 2231.27(-0.03, 6054.57) | 107.22(-0.00, 287.79) | 0.57(0.45, 0.70) | 65.61(-0.00, 187.90) | 3.27(-0.00, 9.23) | 1.39(1.23, 1.55) |
| Ethiopia | 17.50(-0.00, 41.26) | 135.29(-0.00, 319.07) | 0.29(0.09, 0.49) | 0.96(-0.00, 2.42) | 7.46(-0.00, 18.85) | 1.19(1.05, 1.34) |
| Federal Republic of Germany | 1870.02(-0.01, 4130.76) | 61.58(-0.00, 136.00) | 0.98(0.82, 1.15) | 198.50(-0.00, 478.46) | 6.55(-0.00, 15.79) | 2.25(2.09, 2.41) |
| Federated States of Micronesia | 6180.74(-0.06, 14666.07) | 74.38(-0.00, 176.04) | 0.95(0.84, 1.05) | 185.63(-0.00, 475.78) | 2.32(-0.00, 5.89) | 1.60(1.54, 1.67) |
| Federative Republic of Brazil | 2711.07(-0.08, 5862.55) | 62.70(-0.00, 135.51) | -1.11(-1.29, -0.93) | 264.65(-0.01, 633.78) | 6.18(-0.00, 14.90) | 0.44(0.26, 0.62) |
| French Republic | 6018.08(-0.10, 13437.13) | 59.75(-0.00, 133.44) | -0.42(-0.52, -0.32) | 491.26(-0.00, 1182.78) | 4.87(-0.00, 11.72) | 0.72(0.61, 0.83) |
| Gabon | 678.00(-0.01, 1544.32) | 64.60(-0.00, 147.51) | -0.79(-0.93, -0.65) | 99.83(-0.00, 243.68) | 9.25(-0.00, 22.63) | 1.76(1.43, 2.09) |
| Gambia | 5681.26(-0.11, 12379.77) | 53.78(-0.00, 117.04) | -2.03(-2.20, -1.86) | 563.90(-0.01, 1347.27) | 5.41(-0.00, 13.03) | -0.25(-0.55, 0.05) |
| Georgia | 8393.80(-0.10, 20955.74) | 44.05(-0.00, 109.99) | 0.42(0.33, 0.51) | 383.21(-0.00, 1041.21) | 2.03(-0.00, 5.50) | 1.45(1.30, 1.61) |
| Ghana | 8622.28(-0.04, 23214.77) | 31.79(-0.00, 85.26) | -0.11(-0.66, 0.44) | 231.29(-0.00, 666.94) | 0.89(-0.00, 2.52) | 0.40(-0.19, 1.00) |
| Grand Duchy of Luxembourg | 3450.73(-0.05, 7550.41) | 59.53(-0.00, 130.36) | -2.96(-3.07, -2.85) | 405.99(-0.00, 968.30) | 7.13(-0.00, 17.17) | -0.57(-0.85, -0.28) |
| Greenland | 386.09(-0.02, 953.23) | 79.46(-0.00, 194.36) | 0.48(0.43, 0.53) | 11.44(-0.00, 30.59) | 2.45(-0.00, 6.44) | 1.09(1.01, 1.17) |
| Grenada | 48.16(-0.00, 111.77) | 106.36(-0.00, 247.44) | 0.09(0.00, 0.17) | 2.20(-0.00, 5.45) | 4.83(-0.00, 11.99) | 0.44(0.37, 0.50) |
| Guam | 2991.36(-0.04, 7069.57) | 51.09(-0.00, 120.62) | 0.83(0.65, 1.00) | 135.92(-0.00, 346.27) | 2.34(-0.00, 5.95) | 1.68(1.46, 1.90) |
| Guinea | 4394.42(-0.02, 9828.63) | 46.88(-0.00, 104.79) | 1.16(0.93, 1.38) | 221.31(-0.00, 545.50) | 2.36(-0.00, 5.81) | 2.61(2.36, 2.87) |
| Guinea-Bissau | 26779.90(-0.26, 61894.06) | 61.04(-0.00, 140.85) | 2.29(1.92, 2.66) | 1864.80(-0.01, 4767.09) | 4.44(-0.00, 11.24) | 3.93(3.60, 4.26) |
| Hashemite Kingdom of Jordan | 1541.31(-0.00, 3614.22) | 46.05(-0.00, 107.94) | 1.90(1.71, 2.10) | 121.55(-0.00, 315.91) | 3.64(-0.00, 9.45) | 4.04(3.65, 4.42) |
| Hellenic Republic | 385.86(-0.00, 1007.32) | 98.15(-0.00, 254.72) | 1.84(1.72, 1.97) | 13.66(-0.00, 38.57) | 3.59(-0.00, 9.98) | 3.65(3.48, 3.82) |
| Hungary | 1826.69(-0.04, 4652.27) | 86.70(-0.00, 219.53) | 0.86(0.74, 0.98) | 46.59(-0.00, 125.15) | 2.32(-0.00, 6.20) | 1.30(1.22, 1.38) |
| Independent State of Papua New Guinea | 699.33(-0.00, 1580.73) | 54.18(-0.00, 122.51) | -1.87(-2.07, -1.67) | 69.34(-0.00, 166.95) | 5.28(-0.00, 12.77) | 0.15(0.03, 0.26) |
| Independent State of Samoa | 400.82(-0.01, 1059.27) | 105.61(-0.00, 277.36) | 1.78(1.32, 2.24) | 11.81(-0.00, 33.10) | 3.22(-0.00, 8.89) | 2.01(1.68, 2.33) |
| India | 18438.24(-2.78, 42230.67) | 58.51(-0.00, 133.86) | -0.06(-0.23, 0.12) | 542.92(-0.10, 1309.19) | 1.77(-0.00, 4.24) | 1.05(0.82, 1.27) |
| Ireland | 674.18(-0.00, 1596.39) | 142.31(-0.00, 337.10) | 0.65(0.51, 0.80) | 20.90(-0.00, 53.08) | 4.48(-0.00, 11.34) | 0.44(0.28, 0.60) |
| Islamic Republic of Iran | 2819.19(-0.02, 6208.05) | 49.80(-0.00, 109.85) | -1.81(-1.88, -1.74) | 461.32(-0.00, 1125.61) | 8.06(-0.00, 19.82) | 0.37(0.16, 0.58) |
| Jamaica | 41375.92(-1.01, 90311.01) | 64.64(-0.00, 141.20) | -1.55(-1.69, -1.41) | 6295.92(-0.15, 15313.98) | 10.07(-0.00, 24.69) | 0.93(0.75, 1.12) |
| Japan | 694.02(-0.02, 1723.15) | 99.53(-0.00, 245.69) | 0.39(0.27, 0.52) | 23.42(-0.00, 61.68) | 3.44(-0.00, 8.96) | 1.33(1.23, 1.44) |
| Kenya | 189.14(-0.00, 480.08) | 27.66(-0.00, 70.16) | 1.14(0.87, 1.42) | 5.77(-0.00, 15.57) | 0.87(-0.00, 2.34) | 1.58(1.29, 1.87) |
| Kingdom of Bahrain | 3258.81(-0.02, 7141.17) | 109.12(-0.00, 238.76) | 0.00(-0.26, 0.26) | 175.53(-0.00, 417.59) | 5.84(-0.00, 13.92) | 0.35(0.11, 0.60) |
| Kingdom of Belgium | 57110.05(-0.95, 124495.16) | 64.37(-0.00, 140.10) | -1.76(-1.87, -1.66) | 7572.05(-0.11, 18251.01) | 8.41(-0.00, 20.31) | 0.32(0.14, 0.50) |
| Kingdom of Cambodia | 8149.19(-0.01, 19922.21) | 67.46(-0.00, 165.10) | 0.71(0.63, 0.79) | 261.43(-0.00, 689.38) | 2.23(-0.00, 5.83) | 1.44(1.33, 1.54) |
| Kingdom of Denmark | 7721.27(-0.13, 16656.13) | 70.42(-0.00, 151.63) | -1.12(-1.26, -0.98) | 899.18(-0.01, 2138.59) | 8.25(-0.00, 19.66) | -0.16(-0.28, -0.04) |
| Kingdom of Norway | 23.91(-0.00, 56.49) | 58.71(-0.00, 138.82) | -2.32(-2.46, -2.18) | 1.49(-0.00, 3.81) | 3.71(-0.00, 9.48) | -0.88(-0.99, -0.77) |
| Kingdom of Spain | 66.69(-0.00, 150.48) | 103.25(-0.00, 233.41) | 0.77(0.51, 1.02) | 3.36(-0.00, 8.21) | 5.23(-0.00, 12.75) | 1.61(1.42, 1.80) |
| Kingdom of Sweden | 61.57(-0.00, 141.27) | 57.54(-0.00, 131.55) | 0.53(0.33, 0.74) | 3.03(-0.00, 7.76) | 2.82(-0.00, 7.19) | 0.63(0.41, 0.85) |
| Kingdom of Thailand | 2123.64(-0.00, 4799.85) | 31.23(-0.00, 70.70) | 1.84(1.54, 2.15) | 114.68(-0.00, 288.21) | 1.69(-0.00, 4.24) | 3.26(2.93, 3.59) |
| Kingdom of the Netherlands | 2109.76(-0.00, 5124.56) | 54.38(-0.00, 132.31) | 1.86(1.75, 1.97) | 55.46(-0.00, 143.05) | 1.47(-0.00, 3.77) | 2.29(2.19, 2.38) |
| Kingdom of Tonga | 464.16(-0.00, 1155.30) | 79.91(-0.00, 198.86) | 0.92(0.90, 0.94) | 11.49(-0.00, 30.62) | 2.08(-0.00, 5.50) | 1.41(1.37, 1.44) |
| Kyrgyz Republic | 288.52(-0.00, 706.38) | 72.03(-0.00, 176.15) | 1.12(0.84, 1.39) | 10.25(-0.00, 27.59) | 2.59(-0.00, 6.94) | 1.59(1.31, 1.86) |
| Lao People's Democratic Republic | 4575.77(-0.01, 12019.10) | 87.48(-0.00, 229.66) | 0.61(0.45, 0.77) | 119.56(-0.00, 340.08) | 2.38(-0.00, 6.69) | 1.10(0.94, 1.26) |
| Lebanon | 1943.56(-0.01, 4788.73) | 48.21(-0.00, 118.34) | 1.60(1.44, 1.76) | 94.66(-0.00, 242.23) | 2.36(-0.00, 6.02) | 2.53(2.37, 2.69) |
| Lesotho | 6813.76(-0.12, 14790.52) | 72.09(-0.00, 156.58) | -1.50(-1.65, -1.35) | 577.48(-0.01, 1382.01) | 6.12(-0.00, 14.69) | 0.13(-0.11, 0.37) |
| Liberia | 156.04(-0.00, 340.71) | 54.81(-0.00, 119.45) | -1.88(-2.00, -1.77) | 22.76(-0.00, 55.14) | 7.95(-0.00, 19.34) | -0.41(-0.53, -0.28) |
| Libya | 128206.60(-5.10, 298936.03) | 17.57(-0.00, 40.93) | 1.39(1.14, 1.65) | 4834.83(-0.19, 12120.54) | 0.67(-0.00, 1.67) | 2.56(2.26, 2.86) |
| Madagascar | 83431.58(-6.44, 216581.62) | 50.74(-0.00, 132.20) | 1.03(0.91, 1.15) | 2988.73(-0.24, 8048.68) | 1.85(-0.00, 4.97) | 1.94(1.78, 2.10) |
| Malawi | 17546.98(-4.96, 37923.38) | 33.61(-0.00, 72.74) | 1.46(1.16, 1.77) | 2191.67(-0.65, 5202.99) | 4.23(-0.00, 10.03) | 3.25(2.92, 3.59) |
| Malaysia | 8161.91(-0.05, 20645.81) | 45.99(-0.00, 116.45) | 0.43(0.09, 0.77) | 642.59(-0.00, 1727.88) | 3.74(-0.00, 10.01) | 2.49(2.01, 2.98) |
| Mali | 2298.37(-0.02, 5025.15) | 57.60(-0.00, 125.89) | -2.28(-2.38, -2.17) | 334.36(-0.00, 815.21) | 8.35(-0.00, 20.39) | 0.12(-0.06, 0.30) |
| Mauritania | 3761.88(-0.09, 8208.03) | 59.99(-0.00, 130.83) | -2.35(-2.54, -2.15) | 435.51(-0.01, 1064.84) | 6.95(-0.00, 17.02) | -0.34(-0.58, -0.10) |
| Mauritius | 39493.98(-21.10, 84365.92) | 59.58(-0.03, 126.84) | -1.90(-1.97, -1.83) | 5497.11(-3.33, 13096.98) | 8.49(-0.00, 20.32) | -0.25(-0.45, -0.05) |
| Monaco | 1711.33(-0.01, 3917.75) | 100.57(-0.00, 230.27) | 1.00(0.67, 1.32) | 97.05(-0.00, 236.95) | 5.73(-0.00, 13.99) | 1.63(1.35, 1.92) |
| Mongolia | 48656.69(-18.20, 104993.54) | 37.70(-0.01, 80.90) | 0.55(0.36, 0.73) | 8408.96(-3.35, 20067.40) | 6.11(-0.00, 14.63) | 1.94(1.75, 2.12) |
| Montenegro | 2746.84(-0.02, 6481.86) | 51.08(-0.00, 120.43) | -0.11(-0.49, 0.27) | 274.40(-0.00, 692.48) | 5.33(-0.00, 13.31) | 2.43(2.02, 2.84) |
| Morocco | 5710.55(-0.05, 12791.77) | 52.57(-0.00, 117.83) | -1.38(-1.75, -1.02) | 328.93(-0.00, 797.09) | 3.05(-0.00, 7.39) | -0.02(-0.19, 0.14) |
| Mozambique | 11949.81(-3.60, 28275.87) | 73.15(-0.02, 171.74) | 1.88(1.79, 1.97) | 378.24(-0.12, 913.94) | 2.36(-0.00, 5.66) | 2.23(2.11, 2.35) |
| Namibia | 60.73(-0.00, 146.26) | 123.04(-0.00, 294.86) | 1.09(1.00, 1.17) | 1.46(-0.00, 3.75) | 3.07(-0.00, 7.79) | 1.11(1.05, 1.17) |
| Nauru | 806.11(-0.01, 1762.68) | 30.16(-0.00, 66.00) | -0.50(-0.98, -0.02) | 114.92(-0.00, 276.90) | 4.64(-0.00, 11.10) | 1.55(1.08, 2.02) |
| Nepal | 1498.57(-0.04, 3318.36) | 47.23(-0.00, 104.37) | -1.73(-1.90, -1.56) | 74.76(-0.00, 184.87) | 2.40(-0.00, 5.91) | -0.63(-0.85, -0.41) |
| New Zealand | 2284.53(-0.03, 5453.32) | 70.52(-0.00, 168.51) | 0.96(0.87, 1.05) | 68.29(-0.00, 176.19) | 2.15(-0.00, 5.51) | 1.97(1.92, 2.02) |
| Niger | 1266.85(-0.00, 2819.85) | 67.89(-0.00, 151.16) | -0.91(-1.15, -0.68) | 100.02(-0.00, 238.05) | 5.14(-0.00, 12.29) | 0.32(0.14, 0.50) |
| Nigeria | 2393.59(-0.06, 5453.95) | 73.42(-0.00, 167.31) | 0.43(0.19, 0.66) | 288.86(-0.00, 710.08) | 8.84(-0.00, 21.74) | 3.02(2.72, 3.33) |
| Niue | 715.00(-0.01, 1815.01) | 106.08(-0.00, 268.70) | 3.04(2.58, 3.50) | 19.01(-0.00, 51.77) | 2.89(-0.00, 7.83) | 2.87(2.52, 3.22) |
| North Macedonia | 680.46(-0.00, 1745.66) | 39.65(-0.00, 101.75) | 0.98(0.68, 1.28) | 20.23(-0.00, 55.86) | 1.22(-0.00, 3.37) | 1.88(1.57, 2.19) |
| Northern Mariana Islands | 1811.56(-0.02, 4361.82) | 46.54(-0.00, 111.91) | 1.51(1.28, 1.73) | 142.57(-0.00, 375.50) | 3.87(-0.00, 10.06) | 2.97(2.63, 3.31) |
| Oman | 1749.82(-0.02, 3964.44) | 65.63(-0.00, 148.75) | -0.60(-0.84, -0.37) | 140.23(-0.00, 340.04) | 5.20(-0.00, 12.64) | 0.21(0.04, 0.39) |
| Pakistan | 300.96(-0.00, 660.78) | 54.14(-0.00, 118.90) | -2.25(-2.41, -2.08) | 39.59(-0.00, 95.00) | 7.18(-0.00, 17.26) | -0.18(-0.43, 0.07) |
| Palau | 6014.88(-0.12, 14607.51) | 67.42(-0.00, 162.97) | 0.03(-0.20, 0.25) | 162.49(-0.00, 431.18) | 1.90(-0.00, 5.00) | 0.49(0.28, 0.71) |
| Palestine | 3242.58(-0.08, 8099.80) | 59.75(-0.00, 148.78) | 2.86(2.76, 2.96) | 90.30(-0.00, 240.95) | 1.72(-0.00, 4.52) | 3.43(3.32, 3.53) |
| People's Democratic Republic of Algeria | 16280.68(-0.03, 37564.97) | 93.51(-0.00, 215.81) | 0.53(0.37, 0.70) | 804.08(-0.00, 2028.34) | 4.62(-0.00, 11.64) | 2.03(1.91, 2.15) |
| People's Republic of China | 40.84(-0.00, 101.17) | 15.74(-0.00, 39.19) | 1.75(1.05, 2.46) | 2.37(-0.00, 6.26) | 0.92(-0.00, 2.42) | 4.30(3.49, 5.12) |
| Plurinational State of Bolivia | 3816.42(-0.04, 9251.51) | 61.85(-0.00, 148.94) | 0.28(0.19, 0.37) | 105.64(-0.00, 272.46) | 1.77(-0.00, 4.53) | 0.86(0.78, 0.93) |
| Portuguese Republic | 293.31(-0.00, 649.97) | 67.79(-0.00, 149.48) | -2.23(-2.34, -2.12) | 34.51(-0.00, 82.95) | 7.77(-0.00, 18.84) | -0.29(-0.43, -0.14) |
| Principality of Andorra | 29.25(-0.00, 78.56) | 113.28(-0.00, 301.42) | 1.10(0.98, 1.23) | 0.79(-0.00, 2.25) | 3.17(-0.00, 8.88) | 1.06(1.00, 1.11) |
| Puerto Rico | 892.84(-0.00, 2131.43) | 64.36(-0.00, 153.32) | 0.37(0.25, 0.50) | 31.60(-0.00, 82.98) | 2.31(-0.00, 6.04) | 1.46(1.29, 1.62) |
| Qatar | 861.35(-0.00, 1866.01) | 87.26(-0.00, 189.15) | 1.54(1.20, 1.88) | 43.28(-0.00, 102.79) | 4.39(-0.00, 10.42) | 2.46(2.10, 2.81) |
| Republic of Albania | 39768.00(-17.80, 86459.69) | 52.98(-0.02, 115.15) | 0.28(0.18, 0.38) | 2948.49(-1.48, 6875.35) | 3.93(-0.00, 9.16) | 1.65(1.50, 1.81) |
| Republic of Armenia | 60.02(-0.00, 147.14) | 124.07(-0.00, 302.90) | 0.78(0.72, 0.84) | 1.76(-0.00, 4.51) | 3.73(-0.00, 9.51) | 1.14(1.08, 1.20) |
| Republic of Austria | 50.04(-0.00, 116.15) | 122.33(-0.00, 284.98) | 0.09(-0.01, 0.19) | 6.57(-0.00, 16.23) | 16.14(-0.00, 40.28) | 1.35(1.19, 1.52) |
| Republic of Azerbaijan | 394.65(-0.00, 893.75) | 23.97(-0.00, 54.12) | -0.07(-0.27, 0.13) | 16.44(-0.00, 41.03) | 1.03(-0.00, 2.55) | 1.11(1.00, 1.22) |
| Republic of Belarus | 455.53(-0.00, 1003.47) | 90.36(-0.00, 199.29) | 0.28(0.07, 0.48) | 39.53(-0.00, 95.14) | 7.76(-0.00, 18.72) | 1.25(1.13, 1.37) |
| Republic of Bulgaria | 8001.68(-0.07, 20203.44) | 37.56(-0.00, 94.60) | 1.90(1.79, 2.00) | 503.11(-0.00, 1378.18) | 2.40(-0.00, 6.53) | 3.14(2.98, 3.29) |
| Republic of Chile | 4978.84(-0.12, 12444.22) | 61.25(-0.00, 153.02) | 2.25(2.14, 2.35) | 128.10(-0.00, 342.27) | 1.64(-0.00, 4.35) | 2.56(2.49, 2.64) |
| Republic of Colombia | 23892.95(-0.14, 56982.01) | 77.75(-0.00, 185.51) | 1.75(1.44, 2.05) | 802.24(-0.00, 2047.59) | 2.63(-0.00, 6.72) | 3.08(2.83, 3.32) |
| Republic of Costa Rica | 1176.67(-0.02, 2918.88) | 127.83(-0.00, 314.47) | 2.12(1.98, 2.26) | 39.51(-0.00, 102.72) | 4.40(-0.00, 11.31) | 2.95(2.85, 3.05) |
| Republic of Croatia | 6.26(-0.00, 17.20) | 152.07(-0.00, 414.15) | 1.16(1.08, 1.24) | 0.19(-0.00, 0.55) | 4.68(-0.00, 13.35) | 1.30(1.19, 1.40) |
| Republic of Cuba | 5157.14(-0.07, 12511.93) | 35.71(-0.00, 86.48) | 0.78(0.48, 1.07) | 178.32(-0.00, 465.10) | 1.24(-0.00, 3.24) | 1.95(1.67, 2.24) |
| Republic of Cyprus | 10792.11(-0.17, 23413.04) | 65.24(-0.00, 141.09) | -2.27(-2.41, -2.12) | 1440.04(-0.03, 3469.62) | 8.76(-0.00, 21.16) | 0.05(-0.17, 0.26) |
| Republic of Ecuador | 2525.14(-0.35, 5443.27) | 63.05(-0.00, 135.69) | -2.04(-2.12, -1.97) | 368.32(-0.05, 888.41) | 8.84(-0.00, 21.32) | -0.41(-0.49, -0.34) |
| Republic of El Salvador | 915.18(-0.00, 2186.41) | 29.55(-0.00, 70.51) | 1.11(0.96, 1.26) | 66.36(-0.00, 173.29) | 2.15(-0.00, 5.61) | 2.66(2.48, 2.83) |
| Republic of Estonia | 1976.36(-0.03, 4917.08) | 35.01(-0.00, 86.48) | 0.31(0.21, 0.41) | 52.65(-0.00, 142.57) | 0.97(-0.00, 2.58) | 0.84(0.76, 0.91) |
| Republic of Fiji | 59337.72(-8.33, 140511.84) | 92.11(-0.01, 217.50) | 2.36(2.13, 2.59) | 1746.07(-0.26, 4499.07) | 2.77(-0.00, 7.09) | 3.04(2.76, 3.32) |
| Republic of Finland | 1.40(-0.00, 3.36) | 124.94(-0.00, 298.72) | 0.36(0.27, 0.45) | 0.05(-0.00, 0.14) | 4.87(-0.00, 12.41) | 0.72(0.61, 0.82) |
| Republic of Guatemala | 1474.39(-0.02, 3344.52) | 83.50(-0.00, 190.02) | -0.36(-0.56, -0.16) | 102.92(-0.00, 249.19) | 5.74(-0.00, 13.94) | 1.26(0.99, 1.52) |
| Republic of Guyana | 30.50(-0.00, 70.88) | 94.96(-0.00, 220.85) | 0.55(0.40, 0.69) | 1.40(-0.00, 3.45) | 4.43(-0.00, 10.90) | 0.43(0.27, 0.60) |
| Republic of Haiti | 2052.35(-1.21, 4385.48) | 42.06(-0.02, 89.77) | -2.56(-2.74, -2.38) | 290.48(-0.17, 684.35) | 5.99(-0.00, 14.17) | -0.24(-0.59, 0.10) |
| Republic of Honduras | 215.40(-0.01, 505.66) | 13.60(-0.00, 32.09) | 0.41(-0.01, 0.83) | 23.32(-0.00, 59.78) | 1.60(-0.00, 4.06) | 2.40(2.09, 2.70) |
| Republic of Iceland | 91173.53(-21.90, 212971.12) | 102.55(-0.02, 239.13) | 0.97(0.75, 1.19) | 2602.04(-0.75, 6439.79) | 3.00(-0.00, 7.37) | 1.63(1.48, 1.77) |
| Republic of Indonesia | 16.68(-0.00, 38.76) | 129.73(-0.00, 301.66) | -0.23(-0.35, -0.11) | 0.65(-0.00, 1.61) | 5.13(-0.00, 12.60) | 0.02(-0.06, 0.11) |
| Republic of Iraq | 1168.79(-0.00, 2706.91) | 65.32(-0.00, 151.27) | 0.02(-0.06, 0.09) | 96.16(-0.00, 238.05) | 5.61(-0.00, 13.82) | 1.76(1.67, 1.85) |
| Republic of Italy | 1327.10(-0.01, 2960.44) | 54.37(-0.00, 121.28) | 1.04(0.92, 1.16) | 134.38(-0.00, 330.74) | 5.52(-0.00, 13.59) | 2.47(2.36, 2.58) |
| Republic of Kazakhstan | 2999.90(-0.01, 7349.55) | 70.96(-0.00, 173.42) | 0.32(0.20, 0.44) | 77.04(-0.00, 199.46) | 1.89(-0.00, 4.87) | 0.09(-0.07, 0.25) |
| Republic of Kiribati | 2293.98(-0.01, 5532.55) | 65.73(-0.00, 158.30) | 1.02(0.83, 1.21) | 112.48(-0.00, 290.51) | 3.25(-0.00, 8.37) | 1.97(1.82, 2.13) |
| Republic of Korea | 8314.11(-0.05, 20001.16) | 42.39(-0.00, 101.89) | -0.62(-0.84, -0.41) | 501.30(-0.00, 1297.29) | 2.57(-0.00, 6.65) | 1.72(1.52, 1.92) |
| Republic of Latvia | 51243.03(-16.30, 113635.29) | 95.06(-0.02, 210.68) | 1.00(0.93, 1.07) | 1777.90(-0.64, 4334.65) | 3.33(-0.00, 8.09) | 1.49(1.39, 1.59) |
| Republic of Lithuania | 25307.59(-13.90, 53372.52) | 70.54(-0.03, 148.73) | -0.68(-0.80, -0.56) | 1896.68(-1.14, 4376.62) | 5.36(-0.00, 12.42) | 1.29(1.13, 1.45) |
| Republic of Maldives | 6029.20(-0.16, 13153.61) | 55.89(-0.00, 121.59) | -1.91(-2.01, -1.81) | 787.58(-0.02, 1902.04) | 7.40(-0.00, 17.95) | 0.45(0.25, 0.65) |
| Republic of Malta | 1818.49(-0.03, 4053.06) | 59.06(-0.00, 131.73) | -0.82(-0.96, -0.69) | 177.06(-0.00, 427.39) | 5.65(-0.00, 13.68) | 0.95(0.79, 1.11) |
| Republic of Moldova | 407.46(-0.00, 958.99) | 46.37(-0.00, 108.73) | -0.47(-0.69, -0.25) | 55.62(-0.00, 138.65) | 7.02(-0.00, 17.28) | 2.50(2.26, 2.75) |
| Republic of Nicaragua | 11566.42(-0.10, 24928.93) | 25.26(-0.00, 54.46) | 0.23(0.14, 0.32) | 1753.87(-0.01, 4305.32) | 3.78(-0.00, 9.32) | 3.35(3.09, 3.62) |
| Republic of Panama | 2159.65(-0.02, 4876.21) | 68.80(-0.00, 155.36) | -0.67(-0.90, -0.44) | 145.80(-0.00, 360.07) | 4.61(-0.00, 11.38) | 0.56(0.32, 0.81) |
| Republic of Paraguay | 13376.65(-0.32, 29457.57) | 74.47(-0.00, 164.06) | -0.11(-0.23, 0.01) | 888.66(-0.02, 2148.64) | 4.97(-0.00, 12.09) | 1.60(1.48, 1.72) |
| Republic of Peru | 87302.51(-39.70, 185024.02) | 70.77(-0.03, 150.13) | -0.54(-0.84, -0.25) | 6907.10(-3.50, 16200.16) | 5.52(-0.00, 12.96) | 1.03(0.88, 1.17) |
| Republic of Poland | 3213.56(-0.04, 7905.96) | 72.07(-0.00, 177.18) | 0.39(0.19, 0.60) | 93.05(-0.00, 245.32) | 2.14(-0.00, 5.63) | 1.51(1.26, 1.75) |
| Republic of Serbia | 40.29(-0.00, 88.82) | 98.08(-0.00, 216.21) | -1.20(-1.45, -0.96) | 2.10(-0.00, 5.08) | 5.18(-0.00, 12.48) | 0.33(0.15, 0.50) |
| Republic of Singapore | 112.08(-0.00, 250.60) | 85.25(-0.00, 190.61) | -1.07(-1.36, -0.77) | 5.88(-0.00, 14.21) | 4.46(-0.00, 10.79) | -0.06(-0.27, 0.15) |
| Republic of Slovenia | 81.05(-0.00, 179.43) | 105.60(-0.00, 233.70) | -0.13(-0.34, 0.09) | 3.80(-0.00, 9.22) | 4.91(-0.00, 11.89) | 0.47(0.26, 0.68) |
| Republic of Suriname | 68.01(-0.00, 166.57) | 76.88(-0.00, 187.89) | 0.60(0.53, 0.68) | 2.39(-0.00, 6.23) | 2.74(-0.00, 7.12) | 0.78(0.69, 0.87) |
| Republic of Tajikistan | 12.77(-0.00, 31.56) | 38.19(-0.00, 95.14) | -1.09(-1.37, -0.81) | 1.83(-0.00, 4.76) | 5.49(-0.00, 14.47) | 0.08(-0.22, 0.39) |
| Republic of the Marshall Islands | 24.37(-0.00, 64.88) | 30.13(-0.00, 79.56) | 1.96(1.84, 2.08) | 0.87(-0.00, 2.44) | 1.10(-0.00, 3.04) | 2.88(2.69, 3.08) |
| Republic of the Philippines | 6554.58(-0.05, 15379.28) | 30.31(-0.00, 71.08) | 1.19(0.91, 1.48) | 560.30(-0.00, 1439.65) | 3.06(-0.00, 7.80) | 3.49(3.32, 3.65) |
| Republic of the Union of Myanmar | 3008.07(-0.01, 7326.78) | 58.30(-0.00, 141.61) | 1.20(1.05, 1.35) | 91.93(-0.00, 240.48) | 1.82(-0.00, 4.75) | 1.77(1.64, 1.91) |
| Republic of Trinidad and Tobago | 7456.38(-0.15, 16449.52) | 91.56(-0.00, 202.20) | -0.70(-0.87, -0.52) | 538.20(-0.01, 1282.21) | 6.66(-0.00, 15.93) | 1.07(0.92, 1.22) |
| Republic of Uzbekistan | 48.52(-0.00, 114.41) | 68.76(-0.00, 162.32) | 0.91(0.59, 1.23) | 2.30(-0.00, 5.86) | 3.28(-0.00, 8.35) | 2.02(1.67, 2.38) |
| Republic of Vanuatu | 934.48(-0.00, 2353.23) | 34.03(-0.00, 85.54) | 2.58(2.39, 2.78) | 26.47(-0.00, 72.51) | 0.99(-0.00, 2.69) | 3.03(2.81, 3.24) |
| Romania | 1808.03(-0.01, 3974.85) | 37.58(-0.00, 82.65) | -1.28(-1.46, -1.09) | 261.42(-0.00, 641.14) | 5.46(-0.00, 13.40) | 1.30(1.07, 1.52) |
| Russian Federation | 3376.06(-0.05, 7505.00) | 68.79(-0.00, 153.15) | -0.74(-0.83, -0.65) | 278.93(-0.00, 672.45) | 5.60(-0.00, 13.56) | 0.96(0.82, 1.11) |
| Rwanda | 1115.07(-0.01, 2478.17) | 49.70(-0.00, 110.46) | -2.00(-2.24, -1.76) | 115.88(-0.00, 280.83) | 5.44(-0.00, 13.27) | 0.07(-0.17, 0.31) |
| Saint Kitts and Nevis | 200.15(-0.00, 505.07) | 75.31(-0.00, 190.06) | 1.77(1.58, 1.96) | 5.24(-0.00, 14.32) | 2.04(-0.00, 5.53) | 1.63(1.39, 1.88) |
| Saint Lucia | 2750.30(-0.21, 6818.16) | 57.90(-0.00, 142.43) | 0.11(0.06, 0.16) | 63.42(-0.00, 171.78) | 1.44(-0.00, 3.83) | 0.36(0.32, 0.40) |
| Saint Vincent and the Grenadines | 26596.74(-14.50, 59368.50) | 93.37(-0.05, 207.86) | 1.40(1.15, 1.65) | 999.66(-0.59, 2349.10) | 3.54(-0.00, 8.29) | 2.04(1.83, 2.26) |
| San Marino | 1617.54(-0.05, 3965.18) | 55.94(-0.00, 136.44) | 0.61(0.35, 0.86) | 43.67(-0.00, 118.36) | 1.58(-0.00, 4.23) | 1.04(0.79, 1.28) |
| Sao Tome and Principe | 21043.79(-0.47, 45994.54) | 46.28(-0.00, 100.96) | -2.28(-2.37, -2.19) | 2913.12(-0.10, 6963.72) | 6.41(-0.00, 15.39) | -0.22(-0.38, -0.06) |
| Saudi Arabia | 2451.57(-0.00, 6454.13) | 16.48(-0.00, 43.35) | 0.93(0.83, 1.03) | 144.34(-0.00, 404.41) | 0.97(-0.00, 2.72) | 2.80(2.64, 2.95) |
| Senegal | 4573.47(-0.04, 12352.11) | 29.64(-0.00, 78.90) | 1.39(1.21, 1.57) | 243.54(-0.00, 697.94) | 1.64(-0.00, 4.64) | 2.52(2.25, 2.80) |
| Seychelles | 218.62(-0.00, 523.75) | 60.58(-0.00, 145.19) | 0.52(0.36, 0.67) | 8.98(-0.00, 23.39) | 2.50(-0.00, 6.51) | 1.20(1.02, 1.39) |
| Sierra Leone | 4193.82(-1.13, 9258.99) | 41.67(-0.01, 92.14) | -1.78(-2.04, -1.51) | 656.09(-0.20, 1595.72) | 6.44(-0.00, 15.78) | -0.24(-0.59, 0.11) |
| Slovak Republic | 4019.15(-0.05, 8810.17) | 44.97(-0.00, 98.27) | -2.28(-2.48, -2.08) | 575.05(-0.00, 1387.05) | 6.43(-0.00, 15.55) | -0.47(-0.86, -0.07) |
| Socialist Republic of Viet Nam | 3397.93(-0.00, 8098.18) | 40.85(-0.00, 97.34) | 0.39(0.22, 0.56) | 309.47(-0.00, 792.22) | 3.81(-0.00, 9.73) | 2.52(2.43, 2.62) |
| Solomon Islands | 11126.98(-0.19, 24224.61) | 52.47(-0.00, 114.33) | 1.11(0.90, 1.32) | 1069.17(-0.02, 2580.00) | 5.06(-0.00, 12.22) | 3.07(2.69, 3.46) |
| Somalia | 1779.28(-0.00, 4515.40) | 41.27(-0.00, 103.80) | -1.17(-1.37, -0.97) | 66.29(-0.00, 177.97) | 1.58(-0.00, 4.21) | -0.80(-1.06, -0.55) |
| South Africa | 36423.91(-0.09, 84076.34) | 65.02(-0.00, 150.37) | 1.23(0.99, 1.46) | 2258.74(-0.00, 5600.35) | 4.05(-0.00, 10.09) | 3.08(2.83, 3.33) |
| South Sudan | 247.79(-0.00, 606.92) | 48.16(-0.00, 118.18) | 1.35(1.11, 1.59) | 7.76(-0.00, 20.89) | 1.51(-0.00, 4.07) | 2.17(1.91, 2.43) |
| State of Israel | 1449.83(-0.00, 3707.21) | 50.68(-0.00, 129.47) | 1.01(0.86, 1.16) | 42.84(-0.00, 117.64) | 1.55(-0.00, 4.23) | 1.55(1.36, 1.75) |
| State of Kuwait | 0.94(-0.00, 2.30) | 119.59(-0.00, 293.51) | 0.29(0.25, 0.34) | 0.03(-0.00, 0.09) | 4.44(-0.00, 11.71) | 0.91(0.82, 0.99) |
| Sudan | 64.81(-0.00, 156.01) | 139.05(-0.00, 334.89) | 0.19(0.12, 0.25) | 2.37(-0.00, 6.10) | 5.13(-0.00, 13.16) | 0.36(0.25, 0.47) |
| Swiss Confederation | 973.97(-0.00, 2263.88) | 94.83(-0.00, 220.55) | 0.12(-0.07, 0.30) | 52.09(-0.00, 129.85) | 5.03(-0.00, 12.58) | 1.32(1.10, 1.54) |
| Syrian Arab Republic | 3154.38(-0.03, 7501.72) | 40.82(-0.00, 97.04) | 0.44(0.33, 0.55) | 319.17(-0.00, 828.58) | 4.15(-0.00, 10.76) | 2.37(2.27, 2.47) |
| Taiwan (Province of China) | 25019.26(-0.15, 57057.43) | 46.59(-0.00, 106.32) | 3.60(2.84, 4.35) | 2643.35(-0.01, 6678.22) | 4.94(-0.00, 12.48) | 6.30(5.45, 7.16) |
| Togo | 1437.94(-0.02, 3311.20) | 53.70(-0.00, 123.47) | 0.19(-0.27, 0.66) | 63.26(-0.00, 159.66) | 2.40(-0.00, 6.05) | 0.93(0.48, 1.38) |
| Tokelau | 6.34(-0.00, 15.52) | 104.56(-0.00, 255.78) | 0.26(0.19, 0.34) | 0.20(-0.00, 0.52) | 3.32(-0.00, 8.64) | 0.71(0.62, 0.80) |
| Tunisia | 10789.15(-0.25, 25847.63) | 101.11(-0.00, 240.80) | 1.03(0.72, 1.34) | 311.42(-0.00, 798.82) | 3.02(-0.00, 7.62) | 1.68(1.46, 1.90) |
| Turkey | 27917.73(-2.14, 66889.29) | 71.56(-0.00, 172.54) | -1.81(-2.07, -1.56) | 1436.08(-0.15, 3751.28) | 3.62(-0.00, 9.55) | -1.15(-1.33, -0.97) |
| Turkmenistan | 1663.38(-0.01, 3948.04) | 49.30(-0.00, 117.27) | 1.02(0.49, 1.54) | 132.42(-0.00, 335.26) | 4.64(-0.00, 11.47) | 2.96(2.45, 3.47) |
| Tuvalu | 38255.83(-15.00, 81979.15) | 62.13(-0.02, 132.86) | -2.57(-2.65, -2.49) | 5130.42(-2.15, 12010.02) | 8.23(-0.00, 19.33) | -0.64(-0.77, -0.50) |
| Uganda | 13280.45(-0.28, 32506.38) | 73.26(-0.00, 178.66) | 0.53(0.38, 0.67) | 391.75(-0.00, 1021.22) | 2.22(-0.00, 5.73) | 1.06(0.89, 1.23) |
| Ukraine | 166453.01(-86.30, 355599.00) | 58.98(-0.03, 126.11) | -2.06(-2.15, -1.98) | 28124.62(-16.50, 66003.07) | 9.61(-0.00, 22.61) | -1.01(-1.11, -0.90) |
| United Arab Emirates | 62.18(-0.00, 154.59) | 80.70(-0.00, 203.05) | -0.70(-0.84, -0.56) | 4.02(-0.00, 10.18) | 4.99(-0.00, 12.82) | 0.08(-0.07, 0.22) |
| United Kingdom of Great Britain and Northern Ireland | 2834.19(-0.04, 6216.59) | 105.70(-0.00, 231.89) | -0.97(-1.05, -0.88) | 193.81(-0.00, 467.07) | 7.16(-0.00, 17.35) | 0.36(0.24, 0.48) |
| United Mexican States | 8799.99(-0.05, 19692.06) | 48.59(-0.00, 108.51) | -0.50(-0.71, -0.30) | 385.54(-0.00, 961.84) | 2.17(-0.00, 5.39) | 0.18(-0.04, 0.39) |
| United Republic of Tanzania | 94.50(-0.00, 226.13) | 78.13(-0.00, 186.35) | 1.28(1.16, 1.41) | 2.51(-0.00, 6.42) | 2.14(-0.00, 5.44) | 1.03(0.90, 1.16) |
| United States of America | 13325.37(-0.05, 30406.86) | 77.38(-0.00, 176.43) | 0.89(0.71, 1.07) | 979.95(-0.00, 2426.05) | 5.69(-0.00, 14.09) | 2.29(2.12, 2.45) |
| United States Virgin Islands | 27431.41(-0.93, 66861.57) | 44.67(-0.00, 108.49) | 0.67(0.64, 0.71) | 1471.43(-0.06, 3799.22) | 2.41(-0.00, 6.20) | 2.33(2.28, 2.38) |
| Yemen | 2971.42(-0.01, 7482.75) | 27.14(-0.00, 68.01) | 2.05(1.86, 2.25) | 137.73(-0.00, 370.88) | 1.31(-0.00, 3.52) | 2.78(2.49, 3.08) |
| Zambia | 6472.06(-0.03, 17494.79) | 113.68(-0.00, 302.43) | 2.05(1.69, 2.40) | 180.29(-0.00, 497.43) | 3.28(-0.00, 8.84) | 2.65(2.22, 3.08) |
| Zimbabwe | 5704.05(-0.03, 13868.27) | 114.49(-0.00, 276.01) | 3.34(2.52, 4.16) | 153.87(-0.00, 395.51) | 3.21(-0.00, 8.15) | 2.88(2.19, 3.58) |

**Abbreviation:** ASR, age-standardized rate; EAPC, estimated annual percentage change; CI, confidence interval; UI, uncertainty interval; SDI, socio-demographic index; YLLs, years of life lost; YLDs, years lived with disability

##
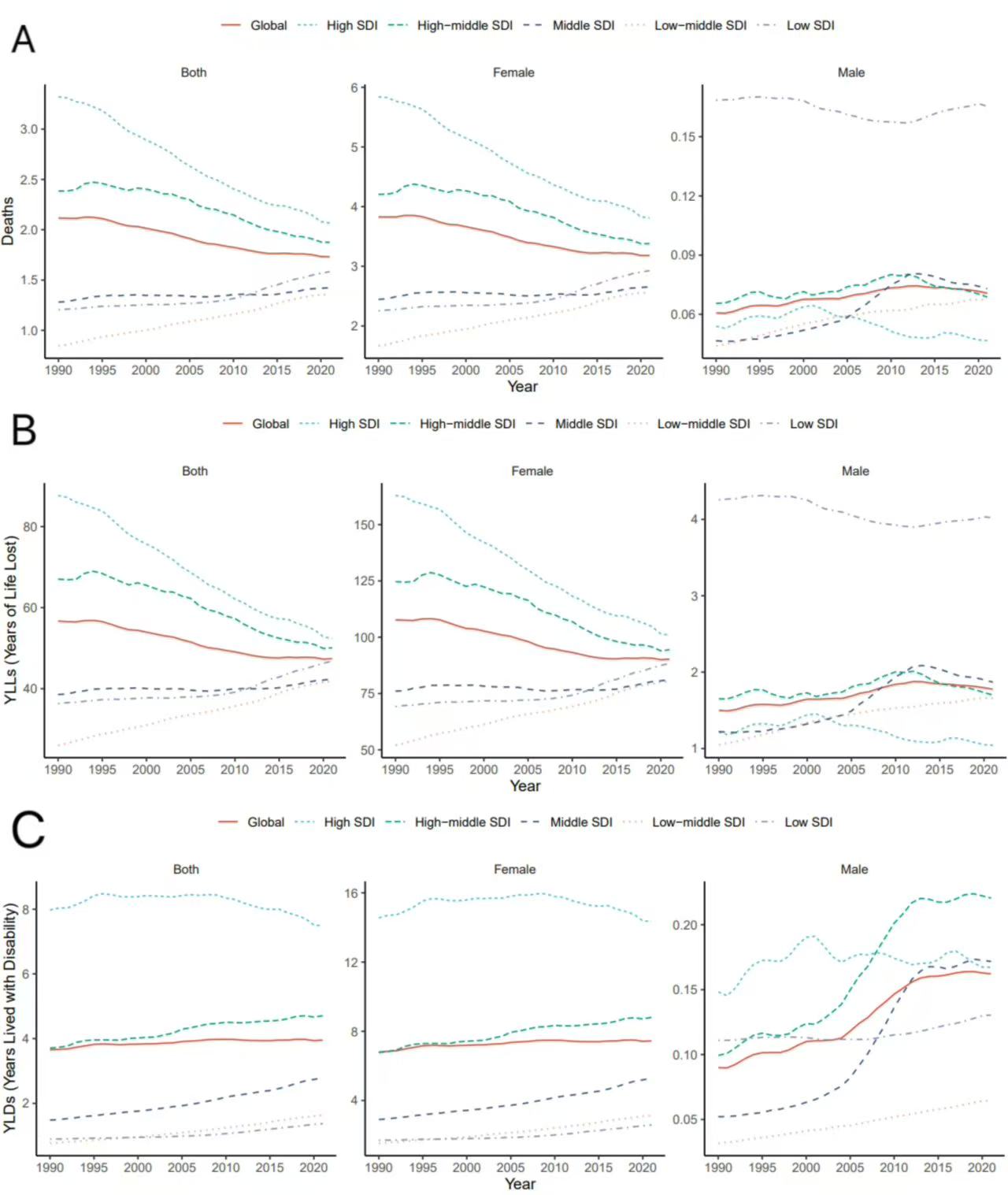
Supplementary Figures

**Figure S1. Trends in the Burden of Breast Cancer Associated with High Red Meat Diets Globally and Across SDI Regions from 1990 to 2021 (By Gender and Total Population).**

From 1990 to 2021, trends in age-standardized mortality rates (A), age-standardized YLLs rates (B), and age-standardized YLDs rates (C) for breast cancer associated with high red meat diets across global and SDI regions, categorized by gender and total population.

**Abbreviations:** SDI, Sociodemographic Index; YLLs, years of life lost; YLDs, years lived with disability.

**
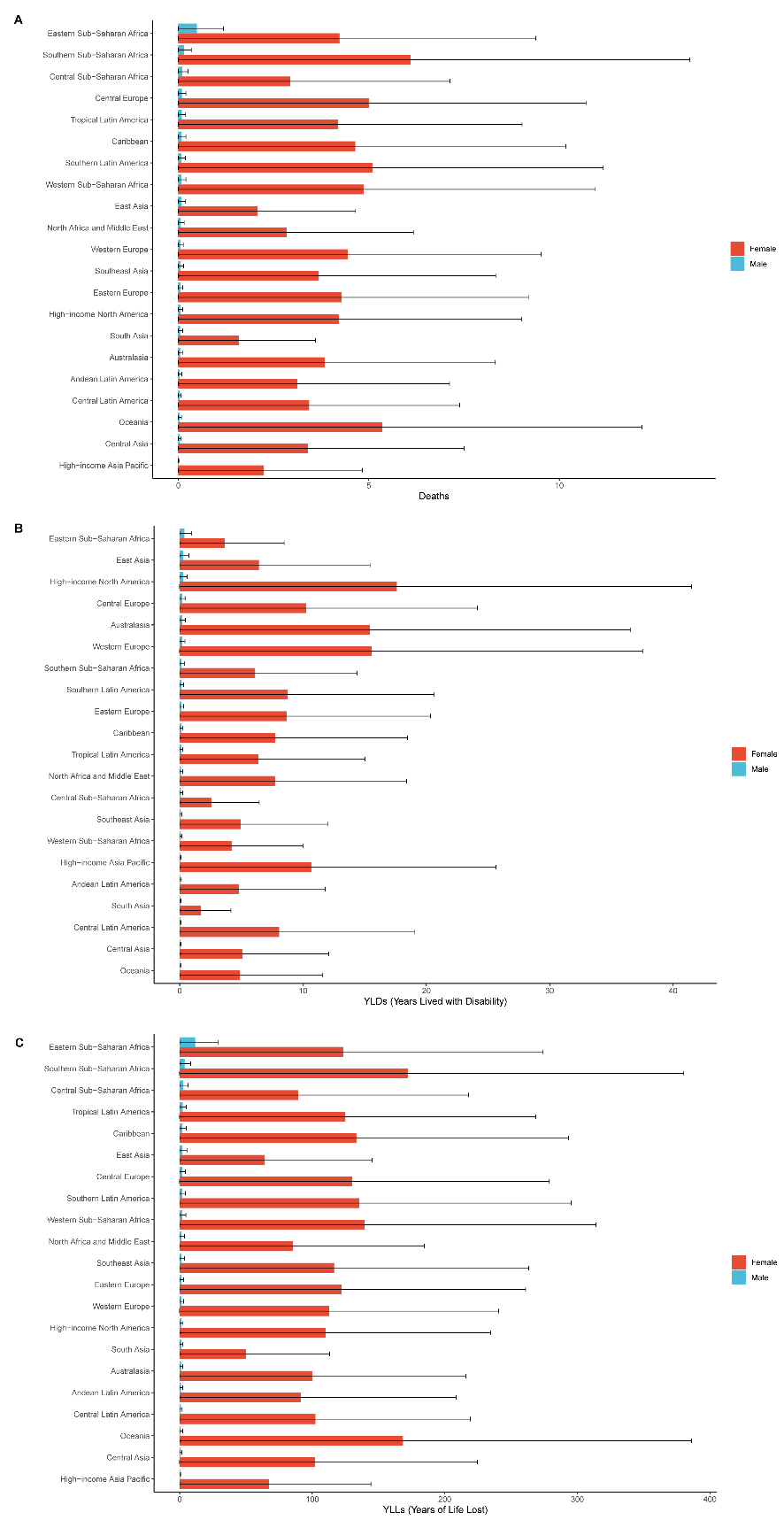
Figure S2. The Burden of Breast Cancer Associated with High Red Meat Diets in 2021 Across Different Regions (By Gender).**

In 2021, age-standardized mortality rates (A), age-standardized YLDs rates (B), and age-standardized YLLs rates (C) for breast cancer associated with high red meat diets across different regions, categorized by gender.

**Abbreviations:** YLLs, years of life lost; YLDs, years lived with disability.

**
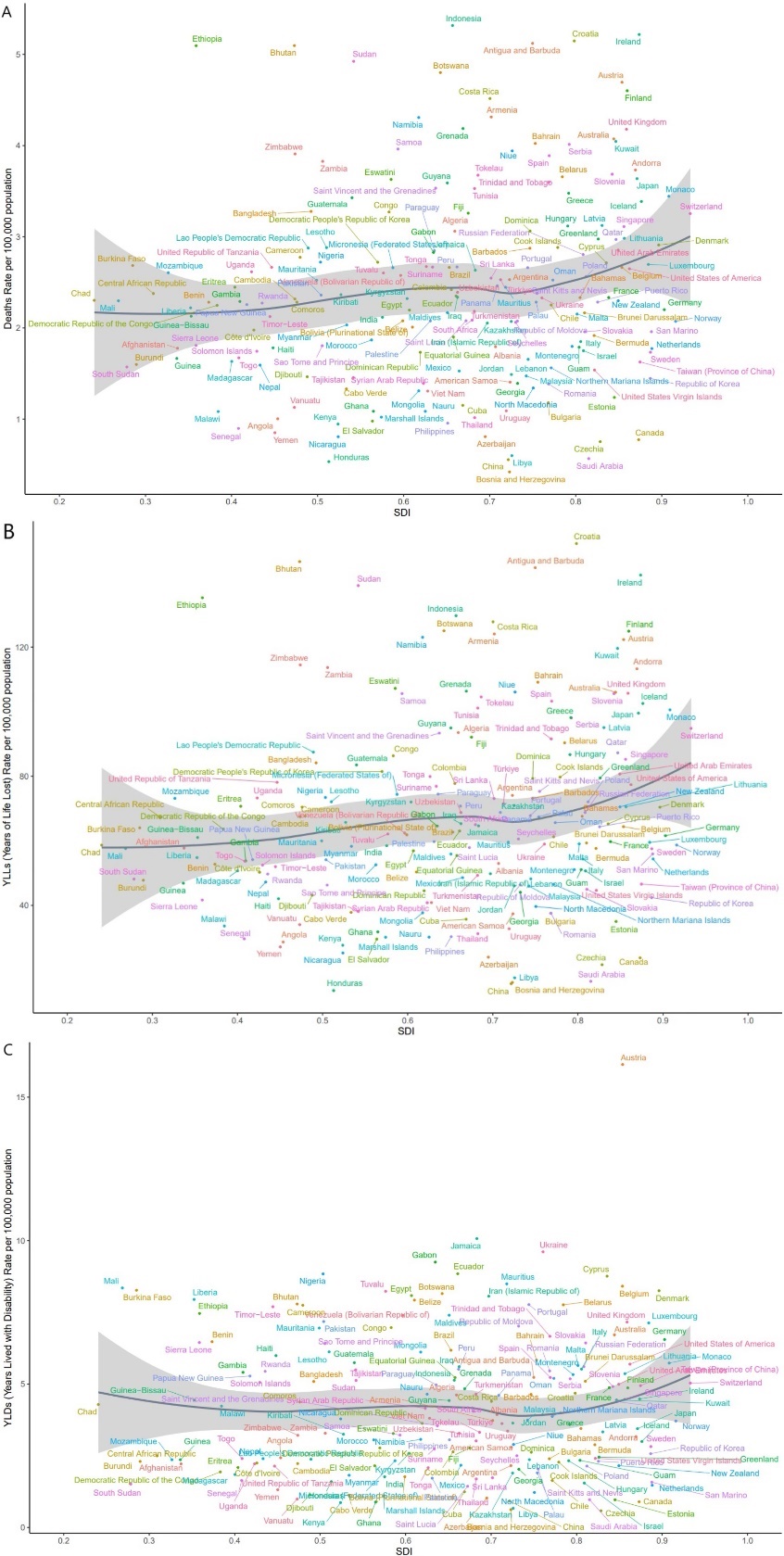
** **Figure S3. The Burden of Breast Cancer Associated with High Red Meat Diets in 204 Countries in 2021.**

Age-standardized mortality rates (A), age-standardized YLLs rates (B), and age-standardized YLDs rates (C) for breast cancer associated with high red meat diets in 204 countries in 2021, and their correlation with SDI regions.

**Abbreviations:** SDI, Sociodemographic Index; YLLs, years of life lost; YLDs, years lived with disability.
